# Facile Rebridging Conjugation Approach to Attain Monoclonal Antibody-Targeted Nanoparticles with Enhanced Antigen Binding and Payload Delivery

**DOI:** 10.1021/acs.bioconjchem.4c00275

**Published:** 2024-09-10

**Authors:** Bayan Alkhawaja, Duaa Abuarqoub, Mohammad Al-natour, Walhan Alshaer, Qasem Abdallah, Ezaldeen Esawi, Malak Jaber, Nour Alkhawaja, Bayan Y. Ghanim, Nidal Qinna, Andrew G. Watts

**Affiliations:** † Faculty of Pharmacy and Medical Sciences, University of Petra, Amman 11196, Jordan; ‡ Cell Therapy Center, University of Jordan, Amman 11942, Jordan; § University of Petra Pharmaceutical Center, Faculty of Pharmacy and Medical Sciences, University of Petra, Amman 11196, Jordan; ∥ Department of Life Sciences, University of Bath, Claverton Down, Bath BA2 7AY, U.K.

## Abstract

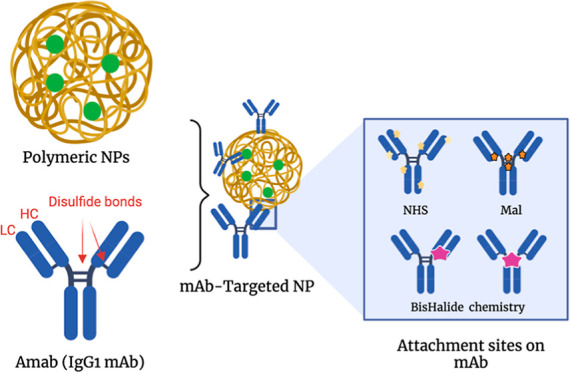

Adopting conventional conjugation approaches to construct antibody-targeted nanoparticles (NPs) has demonstrated suboptimal control over the binding orientation and the structural stability of monoclonal antibodies (mAbs). Hitherto, the developed antibody-targeted NPs have shown proof of concept but lack product homogeneity, batch-to-batch reproducibility, and stability, precluding their advancement toward the clinic. To circumvent these limitations and advance toward clinical application, herein, a refined approach based on site-specific construction of mAb-immobilized NPs will be appraised. Initially, the conjugation of atezolizumab (anti-PDL1 antibody, Amab) with polymeric NPs was developed using bis-haloacetamide (BisHalide) rebridging chemistry, followed by click chemistry (NP-Fab BisHalide Ab and NP-Fc BisHalide Ab). For comparison purposes, mAb-immobilized NPs developed utilizing conventional conjugation methods, namely, *N*-hydroxysuccinimide (NHS) coupling and maleimide chemistry (NP-NHS Ab and NP-Mal Ab), were included. Next, flow cytometry and confocal microscopy experiments evaluated the actively targeted NPs (loaded with fluorescent dye) for cellular binding and uptake. Our results demonstrated the superior and selective binding and uptake of NP-Fab BisHalide Ab and NP-Fc BisHalide Ab into EMT6 cells by 19-fold and 13-fold, respectively. To evaluate the PDL1-dependent cell uptake and the selectivity of the treatments, a blocking step of the PDL1 receptor with Amab was performed prior to incubation with NP-Fab BisHalide Ab and NP-Fc BisHalide Ab. To our delight, the binding and uptake of fluorescent NPs were reduced significantly by 3-fold for NP-Fab BisHalide Ab, demonstrating the PDL1-mediated uptake. Moreover, NP-Fab BisHalide Ab and NP-Fc BisHalide Ab were entrapped with the paclitaxel payload, and their cytotoxicity was evaluated. They showed significant enhancements compared to free paclitaxel and NP-NHS Ab. Overall, this work will provide a facile conjugation method that could be implemented to actively target NPs with a plethora of therapeutic mAbs approved for various malignancies.

## Introduction

**Figure 1:**
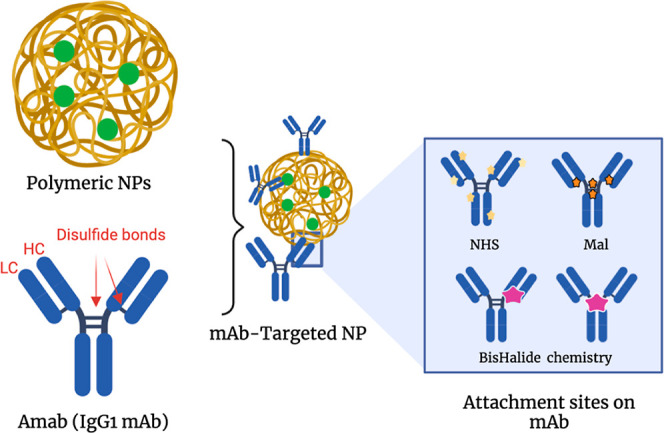
Elaboration of the structures of the targeted NP with mAbs, along with the plausible attachment sites. Conventional *N*-hydroxysuccinimide (NHS) coupling chemistry and maleimide (Mal) chemistries result in heterogeneous immobilized NPs. This work utilizes rebridging chemistry (BisHalide) to construct site-specific and stable targeted NPs. HC: heavy chain, LC: light chain. BisHalide: Bis-haloacetamide compounds created with BioRender.com.

Across the last 3 decades, the advent of monoclonal antibodies (mAbs),^1^ Y-shaped glycoproteins,^2^ has made tremendous advances in the oncology field, cemented by the ever-increasing approval mAbs.^1,3^ As of 2021, more than 100 mAbs have been granted approval and approximately 50% were products for cancer treatment.^4^ Structurally, mAbs comprise two heavy chains and two light chains held together through interchain disulfides (Figure 1). Functionally, the Fab regions are responsible for their intriguing selectivity toward tumor antigens or receptors; following the binding, mAbs interfere and block the associated downstream signaling pathways.^5^ Among tumor antigens, programmed cell death protein 1 (PD1), CD20, and human epidermal growth factor receptor 2 (HER2) are chief players and most widely investigated.^3,6^

The intriguing selectivity for tumor antigens renders mAbs attractive vehicles for tumor drug delivery. Hence, mAb-based drug delivery strategies afford selective means to target tumors while substantially mitigating the side effects encountered by traditional chemotherapeutics. One exemplification of these strategies is what is known as active or targeted nanotechnology.^7,8^ Targeted nanotechnology involves the use of nanoparticles (NPs) that are functionalized with tumor-targeting moieties, such as mAbs.^9,10^ Therefore, they combine both the distinct advantages of being of small size allowing NPs to penetrate biological barriers and functionalization with targeting moieties for widening the therapeutic window of NPs by preferential tumor delivery via tumor antigens and limiting side effects elsewhere.^11^

Following this, various approaches successfully developed targeted NPs functionalized with antibodies and showed promising therapeutic outcomes, notably at the preclinical level.^12,13^ Notwithstanding the promising results, none of the targeted NP approaches has proceeded to clinical approval, which could be attributed to the overlooked immobilization strategies that could fundamentally affect the devoted targeting aims.

Immobilization strategies tend to rely on traditional conjugation methods, encompassing the well-known carbodiimide/NHS coupling reaction between NPs (bearing carboxylic acids) and native lysine moieties of the mAbs.^12,14,15^ Another well-adopted approach is through thiol alkylation of the reduced disulfide bonds of mAbs.^16,17^

Adopting the conventional conjugation approaches exhibited suboptimal control over the orientation of mAb binding or the structural stability of the nanoconstructs. Subsequently, heterogeneous NPs with poor batch-to-batch reproducibility and overall stability are obtained.^18,19^ Therefore, more accessible and reliable immobilization strategies for antibodies are necessitated. To bridge the gap between finding facile conjugation chemistry and developing targeted nanotechnology, we set out to utilize rebridging and click chemistry to retain the superior targeting capabilities of and stability of antibodies.^20,21^ The advantages of using rebridging chemistry are related to maintaining the structural integrity of the antibodies, controlling the orientation of the mAb binding on the surface of the NPs, and translating this approach over most approved anticancer antibodies (IGg1 class of antibodies).

Our group has developed rebridging chemistry using bis-haloacetamide compounds,^22^ and herein, we aimed to implement this approach coupled with click chemistry to construct NP–mAb conjugates. To our knowledge, this is the first report of utilizing rebridging chemistry to immobilize mAbs (native) on the surface of polymeric NPs. All in all, using this accessible platform holds great potential for widening the functionalization of nanocarriers with a plethora of therapeutic mAbs to treat various malignancies.

## Results and Discussion

### mAb Selection Criteria: IgG1 Class of Antibodies

IgG1 is the predominant class of therapeutic mAbs.^23^ Therefore, a model antibody belonging to the superfamily of IgG1 was selected to be immobilized on the surface of NPs. Atezolizumab (Amab), an anti-PDL1 mAb, was selected as the targeting moiety on the surface of polymeric NPs to deliver them toward PDL1-antigen-expressing tumors (Figure S1). Immune checkpoint inhibitors are a new class of antibodies which inhibit the physiological brakes and reactivate the T cell-based attack toward cancer, hence achieving the revival of immunotherapy (Figure S1). The main antigens utilized to activate the T cell response are CTLA-4 and PL1 or PDL1 antigens. T-cell-targeted therapy could be used as monotherapy or in combination with other chemotherapies to treat about 50 malignancies.^24−28^

Previous work has demonstrated the advantages of active targeting of antibodies on the surfaces of NPs. However, conventional fabrication methods might be successful as a proof of concept.^29,30^ Therefore, improving immobilization (conjugation) approaches could be advantageous in proceeding with nanoformulations toward clinical approval.

### Bioconjugation Chemistry for Functionalization of Amab

Therapeutic “ADCs” or antibody–drug conjugates, a new class of mAb-based therapies, are leading a new era in targeted cancer therapy. In principle, ADCs are mAbs linked to cytotoxic drugs through conjugation chemistry to deliver chemotherapeutic drugs to tumors and minimize their toxicity toward normal tissues.^31,32^ ADCs are constructed mainly using the accessible mAb native lysine or cysteine moieties and succinimide- or maleimide-based linkers that are employed to anchor the payload, respectively (Figures 1 and S2).^33^ Using the conventional conjugation methods offered products with minimum control over their homogeneity, batch-to-batch reproducibility, and pharmacokinetic properties. Hence, advanced and site-specific conjugation techniques have been explored to attain ADCs with a reproducible drug-to-antibody ratio (DAR).^34,35^

Previously, our group developed a panel of BisHalide compounds to rebridge the reduced disulfides of mAbs, which permits the introduction of various functionalities while maintaining the integrity of mAbs.^22^ Following ADCs’ footsteps, random immobilization of NPs with mAbs could produce heterogeneous NP products, and their pharmacodynamic and pharmacokinetic properties will be likewise heterogeneous.^36^ Hence, site-specific chemistry, encompassing rebridging and click chemistry, is adopted in this work to control the orientation of the mAbs on the surface of the NP surface.

Following this, the chemical synthesis of the rebridging linkers, namely, linkers **1**, **2**, and **3**, was readily carried out through single- or multisuccessive step procedures with high yields (Experimental Section and Scheme S1). The reactivity of the described linkers toward mAbs has been demonstrated previously.^22^
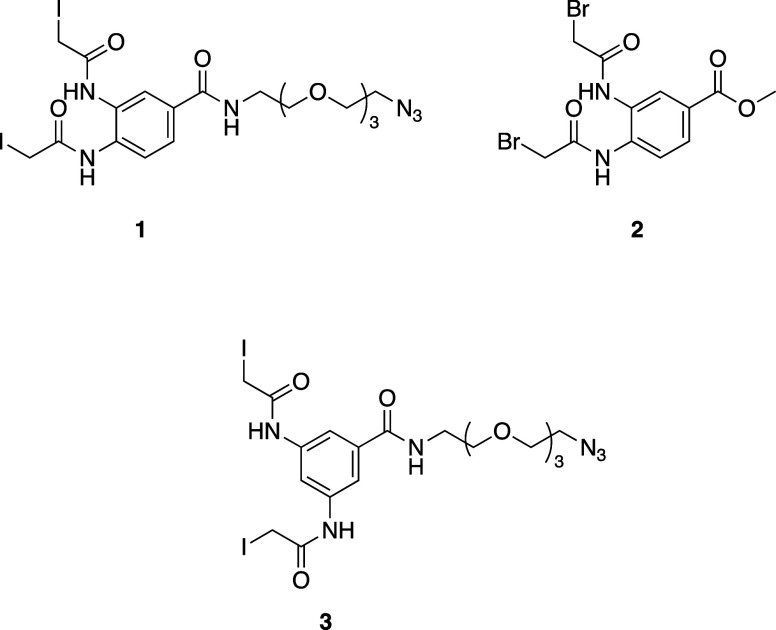


**Figure 2:**
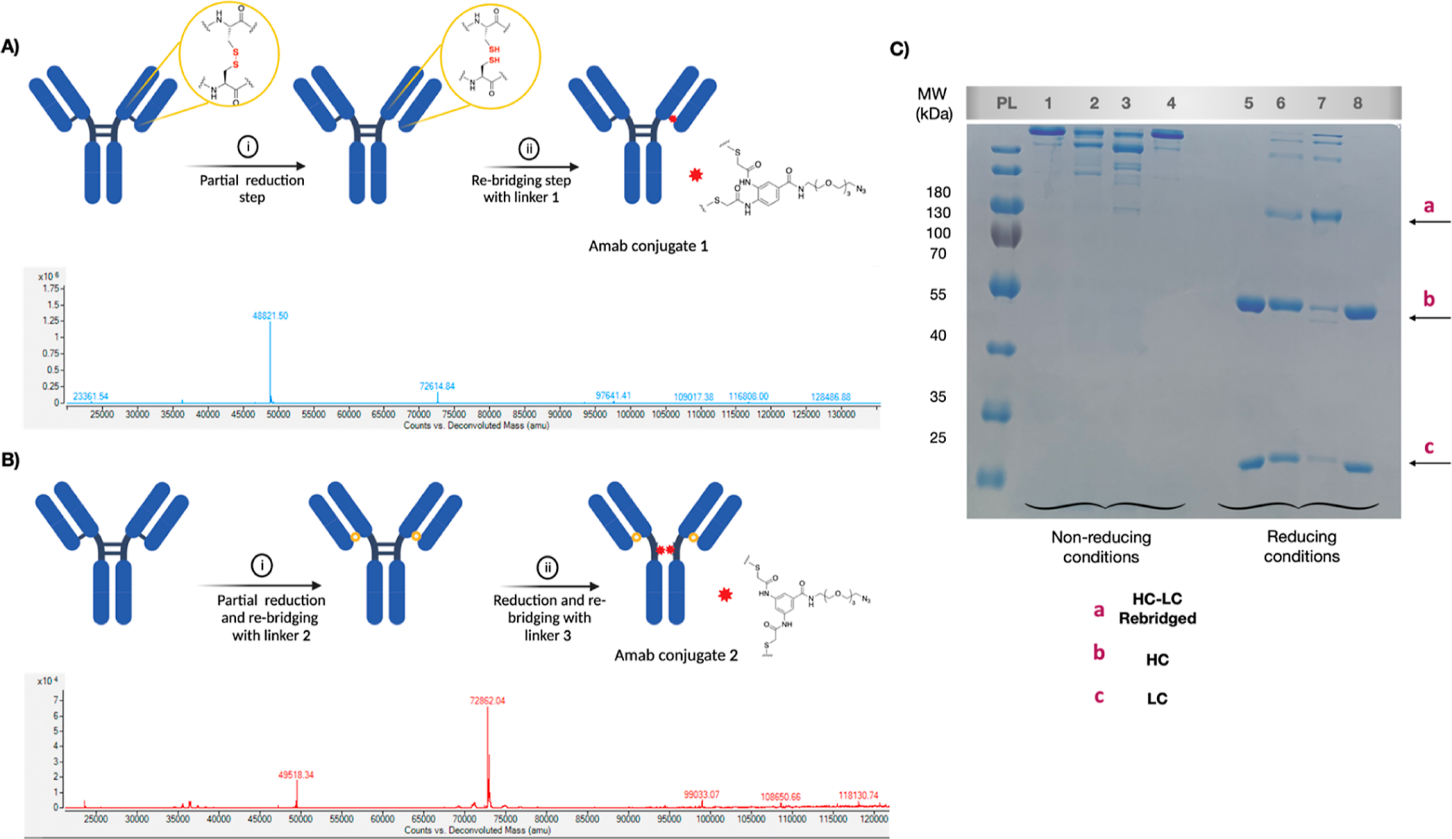
Development and characterization of Amab conjugates. (A) (i) Amab (5 mg/mL) was reduced with TCEP (1.1 equiv) for 2 h at room temperature. (ii) 2.2 equiv of the linker **1** was added to the partially reduced antibody and left to react at room temperature overnight. Deconvoluted spectrum protein mass spectrometry (MS) of partially reduced Amab cross-linked with linker **1** at the HC–LC, showing the peak at 72,614.84 Da. (B) (i) Amab (5 mg/mL) was reduced with TCEP (2.2 equiv) for 2 h at room temperature. Then, 3 equiv of the linker **2** was added and left to react at room temperature overnight. (ii) Blocked Amab (5 mg/mL) was reduced with TCEP (2.2 equiv) for 2 h followed by overnight incubation with linker **3** (5 equiv). Deconvoluted spectrum protein MS of Amab cross-linked with linkers **2** and **3** at the HC–LC and hinge region, respectively, showing a major peak at 72,862.04 Da. (C) Sodium dodecyl sulfate-polyacrylamide gel electrophoresis (SDS-PAGE) analysis of rebridging of reduced Amab (5.0 mg/mL, 34 μM) in Tris-HCl buffer (100 mM, pH 7.5); PL: protein ladder; lane 1: Amab control (NR), lane 2: Amab conjugate 1 (NR), lane 3: Amab conjugate 2 (NR), lane 4: Amab conjugate 3 (NR), and lanes 5–8: similar to 1–4 samples but resolved under reducing conditions. Protein samples were resolved by SDS-PAGE (10% gel). NR: Nonreducing, HC: heavy chain, LC: light chain.

In this work, we intended to develop a conjugation step facilitating the superior orientation of antibodies where the mAbs immobilize through the Fc region, rendering the Fabs available to interact with cellular antigen. To this end, we designed *Amab conjugate 1* (Amab-Fab-N3) and *Amab conjugate 2* (Amab-Fc-N3), where one or both Fabs are freely available for binding to the PDL1 receptor, respectively (Figure 2A,B).

To attain *Amab conjugate 1* (Amab-Fab-N_3_), Amab (5 mg/mL) was reduced with TCEP (1.1 equiv) for 2 h at room temperature, followed by conjugation with linker 1 overnight. The products were characterized using SDD-PAGE and protein MS (Figure 2). The calculated molecular weight (MW) of rebridged heavy and light chains is 72,614, and the found MW was 72,614, as shown in Figure 2A. Given that only one heavy and light chain was cross-linked, the spectrum displayed the unconjugated light chain (23,361.54 Da) and heavy chain (48,821.50 Da).

*Amab conjugate 2* (Amab-Fc-N3), on the other hand, requires the extra step of reduction and labeling with linker **3** after the initial labeling step with linker **2**. Previously, we found that linker **3** preferably rebridges heavy light disulfides; this step was conducted using a limited equivalent of TCEP.^22^ Gratifyingly, MS results displayed the bimodified *Amab conjugate 2* (Amab-Fc-N_3_) as a major product with the found MW of 72,862.04 Da. The attained Amab conjugates were purified via diafiltration into PBS buffer to remove unreacted linkers and stored in a fridge. Conjugate stabilities were confirmed after being held in the refrigerator for up to 3 months, as shown by the MS results (Figure S8).

For comparison reasons, *Amab conjugate 3* (Amab-Mal) was developed using well-known maleimide chemistry. Briefly, Amab (5 mg/mL) was reduced with TCEP (5 equiv) for 2 h at room temperature followed by Michael addition reaction with Maleimide-PEG_3_-N_3_ (20 equiv) and left overnight. The products were characterized using SDD-PAGE and protein MS, showing that LC was modified with one azide group, whereas the HC peak confirmed three attached azide moieties (Figure 2 and Supporting Information).

### Nanoformulation and Amab-Decorated NPs

Eudragit L-100 belongs to the versatile family of methacrylate polymers; it has been enormously employed in formulations for various drug delivery purposes. Eudragit L-100 is an anionic copolymer of methacrylic acid and methyl methacrylic acid and is mainly used for jejunum drug delivery and drug release above pH 6. In addition, tablet formulations, NPs, liposomes, and microspheres were prepared using Eudragit L-100. Therefore, due to their well-established safety profile, Eudragit L-100 was selected to prepare NPs bearing a carboxylic acid group for further activation.^37^

Eudragit L-100 NPs were prepared using the nanoprecipitation protocol; to this end, the NP was loaded with either a fluorescent dye (coumarin 6) or an anticancer drug (paclitaxel, PTX) for further cellular binding or cytotoxicity assays, respectively. Following synthesis, Nude NPs loaded with coumarin 6 (Nude NP) or PTX (referred to as Nude PTX-NP) were activated with carbodiimide/NHS reagents affording NP-NHS and NP-NHS (PTX), respectively. Then, NP-NHS and NP-NHS (PTX) were coupled to primary amines of Amab to attain NP-NHS Ab and NP-NHS Ab (PTX), respectively.

For performing click reaction, NP-NHS was further functionalized with dibenzocyclooctyne-amine; following this, azide-functionalized Amab, *Amab conjugate 3* (Amab-Mal) was clicked with it to afford NP-Mal Ab (Table 1).

**Table 1:** Characterization of Nanoformulations

nanoformulation	Amab conjugate	diameter (nm) ± SD	zeta potential ± SD	PDI
Loaded with coumarin 6				
unconjugated NP		128.7 ± 1.18	–13.7 ± 2.7	0.315
NP-NHS		143.7 ± 1.37	–17.7 ± 0.9	0.195
NP-NHS Ab	native Amab	139.9 ± 1.21	–18.3 ± 0.35	0.253
NP-Mal Ab	Amab-Mal	143.9 ± 1.57	–11.8 ± 1.7	0.209
NP-Fab BisHalide Ab	Amab-Fab-N_3_	138.2 ± 1.11	–20.1 ± 3.3	0.331
NP-Fc BisHalide Ab	Amab-Fc-N_3_	131.13 ± 1.41	–22.9 ± 2.9	0.188
Loaded with PTX				
unconjugated NP(PTX)		150.7 ± 1.19	–15.5 ± 1.12	0.218
NP-NHS (PTX)		145.5 ± 1.35	–20.1 ± 1.8	0.260
NP-NHS Ab (PTX)	native Amab	145.2 ± 1.13	–20.7 ± 2.1	0.269
NP-Mal Ab (PTX)	Amab-Mal	157.5 ± 1.54	–13.6 ± 0.68	0.356
NP-Fab BisHalide Ab (PTX)	Amab-Fab-N_3_	151.3 ± 1.52	–17.7 ± 1.4	0.315
NP-Fc BisHalide Ab (PTX)	Amab-Fc-N_3_	145.5 ± 1.33	–27.2 ± 3.1	0.195

The optimal orientation of antibodies on the surface of NPs is known as end-on, where the mAbs immobilize through the Fc region, rendering the Fabs available to interact with cellular antigen. In contrast, flat-on orientation is suboptimal, where the antibody is linked through one Fc and one Fab fragment, rendering the other Fab available for targeting.^18^

Having successfully rebridged the interchain disulfides of Amab with the azide-functionalised linker, we next set out to selectively decorate Amab to the surface of polymeric nanoparticles. Two targeted NPs using click reaction were developed with NPs bearing the dibenzocyclooctyne group. NP-Fab BisHalide Ab was developed through the Fab region using *Amab conjugate 1* (Amab-Fab-N_3_), affording NP with one free Fab region (flat-on immobilisation) for optimised antibody confirmation. NP-Fc BisHalide Ab was developed by binding to the Fc region using Amab conjugate 2 (Amab-Fc-N3), which is functionalised with an azide group at the hinge region, affording (end-on orientation) for superior antibody orientation at the surface of NPs.

On the other hand, binding through the Fc region (end-on orientation) could afford superior antibody orientation at the surface of NPs. Therefore, we explored our novel chemistry to introduce clickable chemistry at the hinge region and gratifyingly succeeded in attaining *Amab conjugate 2* (Amab-Fc-N_3_). After that, a click reaction was performed with NPs bearing the dibenzocyclooctyne group, affording NP-Fc BisHalide Ab.

Following the synthesis and surface functionalization of NPs, a zetasizer was used to investigate the physicochemical properties of synthesized NPs in terms of hydrodynamic average particle size, polydispersity index (PDI), and zeta potential. The dynamic light scattering results showed a size range between 128.7 and 157.5 nm with reasonable variations in sizes and shapes, as indicated by PDI (Table 1). Interestingly, the dimensions of PTX-loaded NPs were slightly larger than those of the unloaded NPs, which is likely due to the loading of PTX. Moreover, zeta-potential analysis revealed a negative surface charge of both dye- and PTX-loaded NPs, which could be attributed to the free carboxylic acid groups of the Eudragit L-100 polymer (Table 1). Coupling % was calculated and found to range from 60 to 80%, which confirms the surface immobilization of the NPs (Table S1).

### In Vitro Cellular Binding and Uptake of the Fluorescent NPs

**Figure 3:**
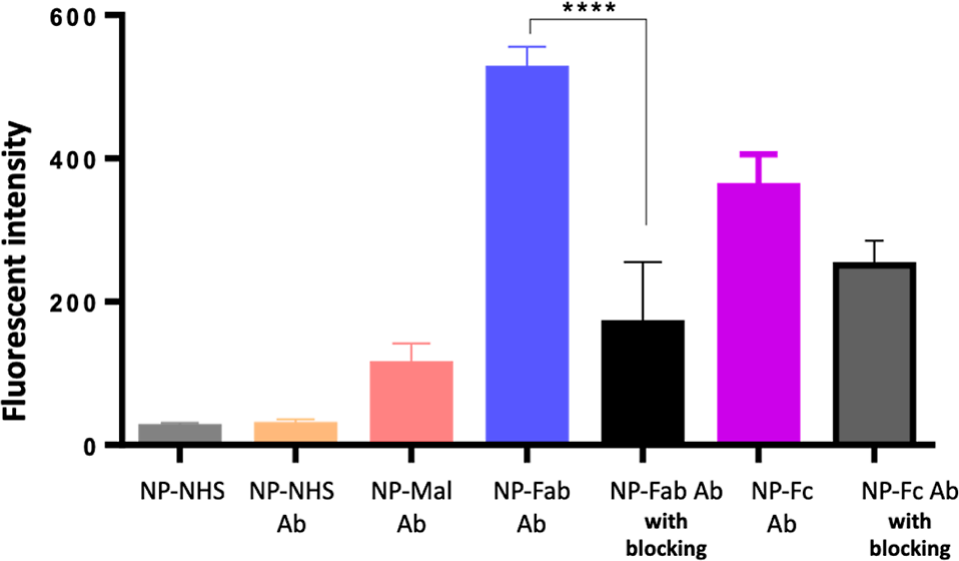
Cellular binding and uptake of the fluorescent nanoformulations. EMT6 cells were treated with the NPs (125 μg polymer/mL) for 45 min; the fluorescent intensity results were displayed as the mean + SD, and the data were subtracted from the control (untreated cells). For the blocking experiment, EMT6 cells were treated with Amab (20 μg/mL) for 15 min at 4 °C prior to the incubation with the nanoformulations.

Initially, NPs loaded with coumarin 6 were evaluated for cell binding and uptake by using flow cytometry and confocal spectroscopy. In this regard, flow cytometric analysis showed that the ability of cells to uptake the NPs conjugated with Amab prepared using BisHalide rebridging chemistry (NP-Fab BisHalide Ab and NP-Fc BisHalide Ab) was significantly higher than all the other treatments, including NP-NHS, NP-NHS Ab, and NP-Mal Ab (*p* value < 0.001). Our results confirmed the higher cellular uptake using our refined conjugation chemistry (Figure 3). Given that NP-Fab BisHalide Ab and NP-Fc Ab were proposed in this work as refined NP conjugates, Fab-based and Fc-based decoration gave optimum cell binding/uptake compared to random binding methods (NP-NHS and NP-Mal).

Random immobilization strategies of targeting moieties, i.e., antibodies using amine (NP-NHS Ab), afforded heterogeneous NP conjugate species due to the high surface abundance of lysine moieties on Amab (more than 80 sites). Hence, there is minimal control over the orientation of the antibodies on the surface of the NPs. Our results have demonstrated significantly lower cellular binding/uptake of NP-NHS Ab and agree with previous findings.^38−40^

On the other hand, the main disadvantages of maleimide chemistry used in the preparation of NP-Mal Ab are related to the random conjugation of antibodies on the surface of NPs and the reduced overall stability of the antibodies. In addition, their reversibility and susceptibility to retro-addition reactions under physiological conditions^41^ render them unsuitable for clinical implications.

One can note that NP-Fab BisHalide Ab exhibited significantly higher fluorescent intensity than NP-Fc BisHalide Ab, reflecting higher cell binding and/or uptake (Figure 3). Hence, NP-Fab BisHalide Ab is considered a candidate nanoformulation with higher cellular uptake.

Next, to confirm that the observed high cell uptake was mediated via the PDL1 receptor, a blocking step with Amab was performed before the binding experiment. To this end, the ability of cells to uptake the NPs conjugated with Amab using BisHalide chemistry was considerably lower compared to the unblocked groups and was found to be significant with NP-Fab BisHalide Ab, confirming the PDL1-mediated cellular uptake (Figure 3 and Table S2). NP-Fc BisHalide Ab was proposed to represent the optimal surface decoration of polymeric NPs (Fc-based binding). Notwithstanding, it gave inferior results to NP-Fab BisHalide Ab regarding PDL1 affinity (without the blocking step) and selectivity (with the blocking step), suggesting that Fab-based immobilization of full mAbs is optimal for PDL1-mediated cell binding/uptake.

It is worth highlighting that the selection of EMT6 cells in this study was intentional to provide a proof-of-concept model that would facilitate future in vivo validation. EMT6 cells allow for the creation of syngeneic allografts in immunocompetent BALB/c mice, enabling an intact immune response that is critical for evaluating the efficacy of PDL1 antibodies.

**Figure 4:**
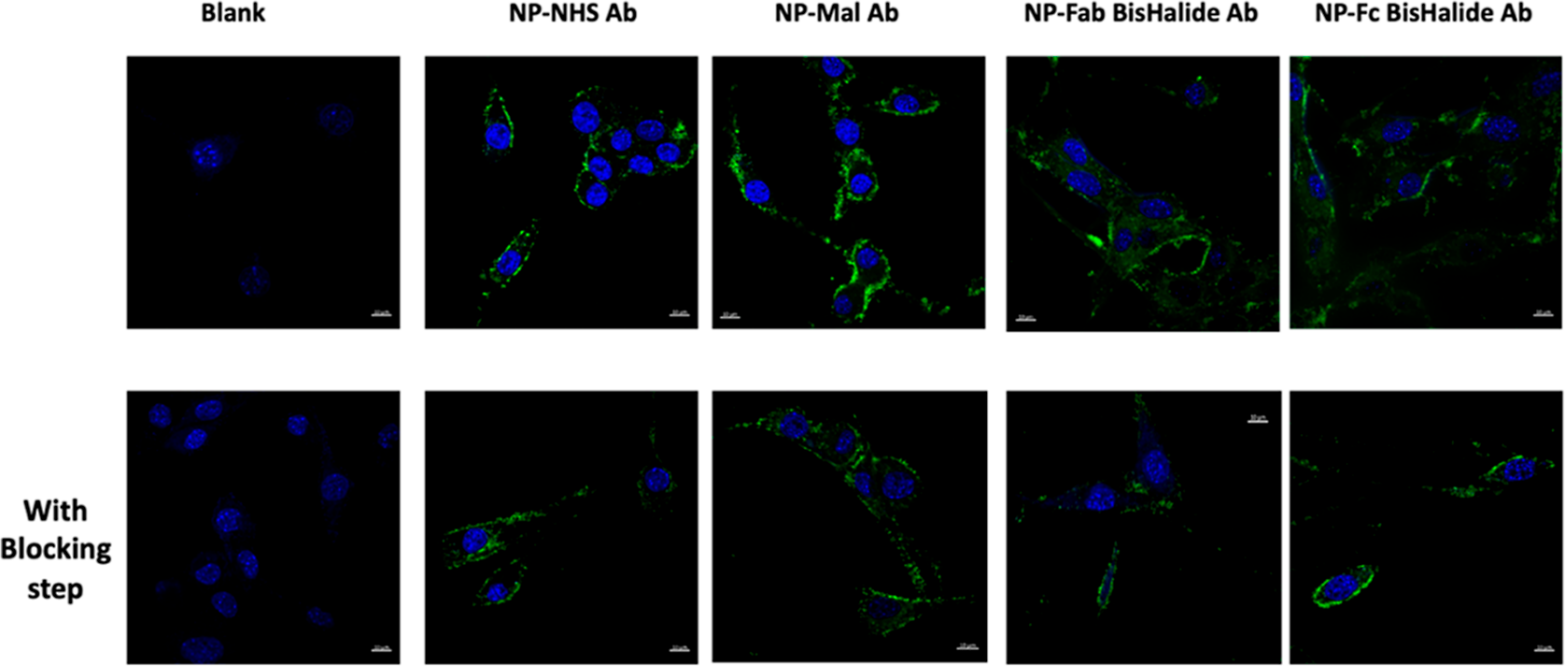
Appraisal of cellular binding and uptake of NP bioconjugates using confocal microscopy. (A) EMT6 cancer cells were treated with fluorescent NP conjugates (green dye) for 45 min. (B) EMT6 cells were blocked with Amab (20 μg/mL) for 15 min at 4 °C prior to the incubation with the nanoformulations separately.

Having demonstrated that higher cellular binding and uptake were achieved through the refined NP conjugates, imaging of the cellular binding and uptake using confocal microscopy was performed. As shown in Figure 4, the site-specific targeting of NPs using BisHalide chemistry facilitated the uptake of the fluorescent NPs into EMT6 cells, as the penetration of the NPs was enhanced compared to NP-NHS Ab and NP-Mal Ab (membrane localization of the NPs) (Figure 4).

### Cellular Toxicity of the PTX-Loaded NPs

Having demonstrated the enhanced cellular uptake of Amab-decorated NPs prepared using BisHalide rebridging chemistry, the effectiveness of this approach to target the cytotoxic payload was evaluated using PTX as a model drug. Following this, NPs loaded with the PTX payload were prepared, followed by conjugation with Amab as described previously. The constructed targeted NPs loaded with PTX [referred to as NPs (PTX)] were characterized as described in Table 1.

**Figure 5:**
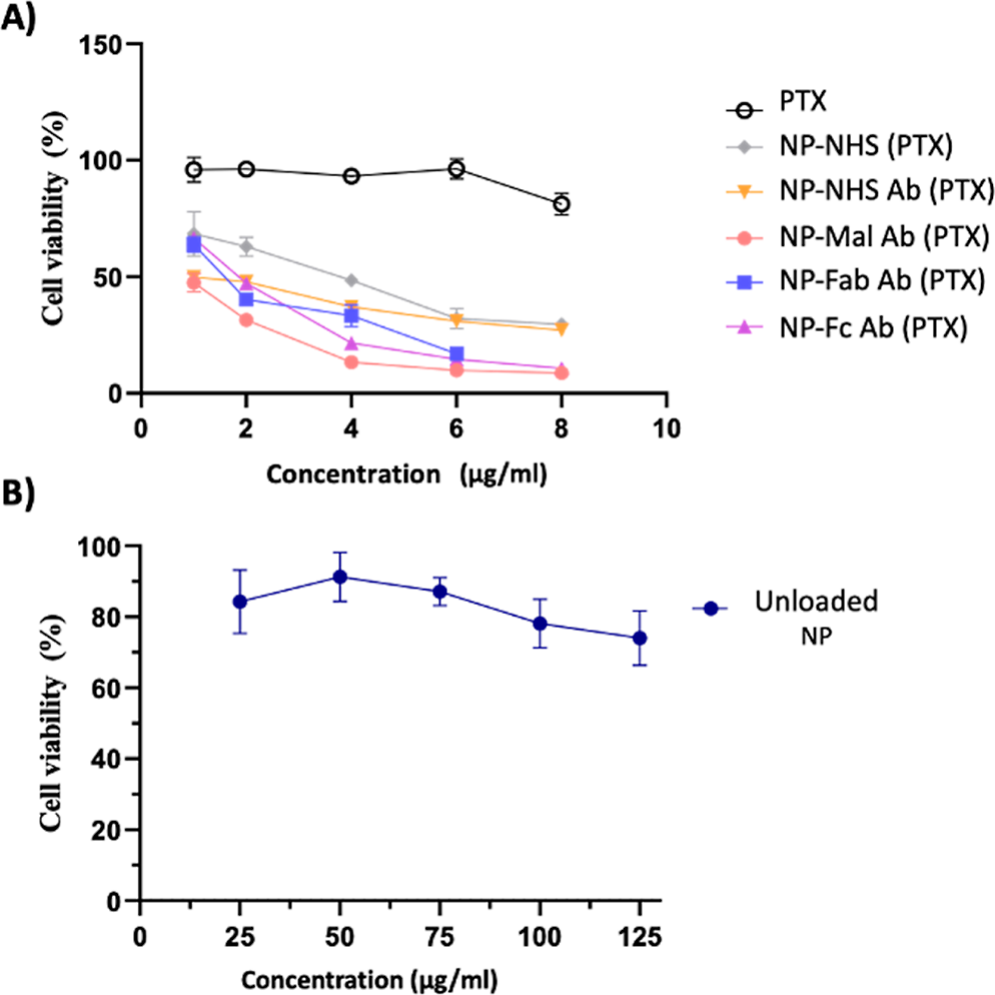
Cytotoxicity of the NPs. (A) Cell viability (%) was performed using the MTT assay. EMT6 cells were treated with PTX-loaded nanoformulations and incubated for 24 h. (B) Unloaded NPs exhibited negligible cytotoxicity toward EMT6 cell lines. The calculated IC_50_ are 3.13, 1.86, 0.84, 1.61, and 1.69 μg/mL for the NP-NHS (PTX), NP-NHS Ab (PTX), NP-Mal Ab (PTX), NP-Fab BisHalide Ab (PTX), and NP-Fc BisHalide Ab (PTX), respectively.

Next, the MTT assay was performed for all nanoformulations and compared with free PTX and unloaded NPs. One can infer that labeling with Amab gave dose-dependent cell toxicity with comparable results among all treatments, with significantly lower cell viability than free PTX and unlabeled NPs [NP-NHS (PTX)] (Figure 5A and Table S3). PTX is a potent cytostatic agent known to induce cell cycle arrest at the G2/M phase and subsequent apoptosis in a dose- and time-dependent manner.^42^ This implies that if PTX is not retained within the cells, it will likely be washed out before exerting its effect. This cytotoxicity assay had a 45 min exposure period to PTX, followed by a 24 h drug-free recovery period. The absence of significant changes in cell viability upon exposure to nonformulated PTX within this time frame is consistent with the expected kinetics of apoptosis induction by PTX.

NP-Fab BisHalide Ab and NP-Fc BisHalide Ab exhibited improved cytotoxicity compared to NP-NHS Ab (Figure 5 and Table S3). It is worthwhile to mention that control nanoformulations (unloaded) with equivalent polymer concentrations were evaluated and exhibited negligible cytotoxicity (Figure 5B).

On the other hand, NP-Mal Ab showed a remarkable reduction in cell viability compared to the other PTX-loaded NPs (Table S3). Although NP-Mal Ab exhibited lower cellular viability than other PTX NPs, using maleimide-based chemistry to attain nanoformulation afforded heterogeneous species of Amab-decorated NPs. Moreover, researchers reported the plasma instability of maleimide-based ADCs,^43^ therefore, the questionable stability of this nanoformulation is another drawback for widespread applications.

**Figure 6:**
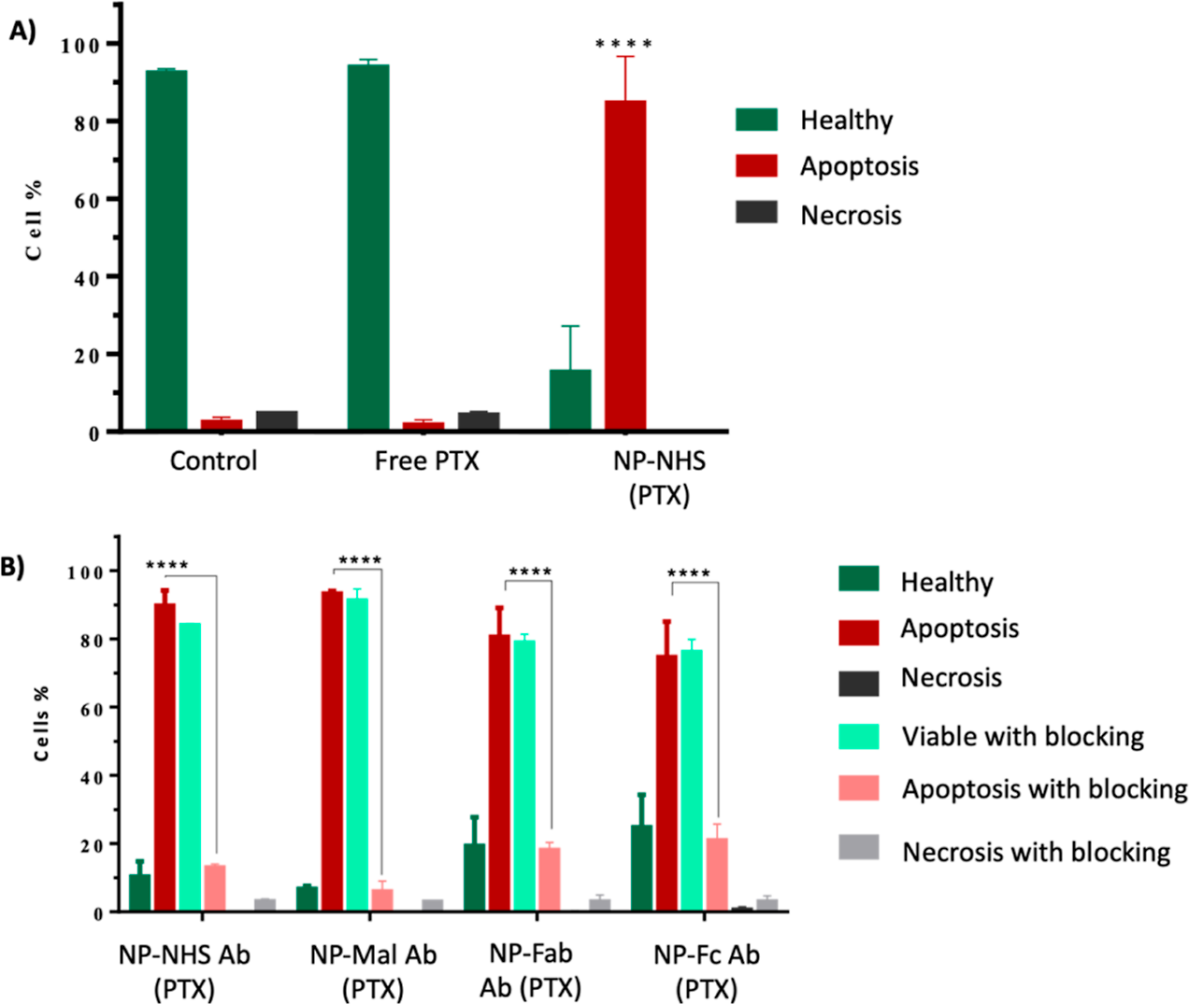
Cytotoxicity of the PTX-loaded NPs. Cell death modality was performed using the Annexin V/PI assay. EMT6 cells were treated with free PTX (32 μg/mL) or Amab (20 μg/mL) for 15 min at 4 °C where appropriate (blocking step), followed by the incubation with the various nanoformulations (equivalent PTX concentration) for 45 min. (A) Untargeted treatments. (B) Targeted PTX-NP treatments.

Next, to evaluate cell death modality and examine whether it is mainly related to apoptosis, we performed an Annexin V/propidium iodide (PI) assay. Apoptosis was found to be the main mechanism of death in all of the treatments. Moreover, our results showed that the number of viable cells decreased significantly in a statistical manner for all the nanoformulations (*p* value < 0.0001) in comparison to the control (untreated) group. Clearly, free PTX exhibited substantially less cell death compared to the NP-loaded nanoformulations (Figure 6A and Table S3), whereas labeling with Amab facilitated the internalization and cell uptake of the nanoformulations through the engagement with PDL1 receptors in all the labeled NPs (NP 3, 4, 5 and 6), as the vast majority of the cells were apoptotic (Figure 6B).

Lastly, to confirm that the observed apoptosis was mainly mediated through PDL1 receptor engagement, the blocking step was performed before incubation of the targeted NP conjugates. Blocking with Amab showed a higher percentage of viable cells when compared to the no-blocking group (*p* value < 0.0001), which demonstrates the selective toxicity of the targeted NPs (Figure 6B).

To our knowledge, this work provided the first translation of next-generation ADC approaches and site-selective rebridging conjugation chemistries to craft mAbs on the surface of NPs. Collectively, as described in this work, active targeting of NPs with mAbs has improved the selective uptake and cytotoxicity of the NPs. Moreover, site-specific conjugation of the mAbs on the surface of the NPs has afforded a more reliable approach for labeling NPs. The random carbodiimide/NHS-based coupling gave inferior selectivity, cellular uptake (internalization), and cytotoxicity compared to the bis-haloacetamide rebridging approach described in this work. More importantly, the provided adaptable conjugation and labeling methods could be translated to target NPs with various mAbs and hence could be implemented to treat different types and subtypes of malignancies.

## Experimental Section

### Chemistry General Remarks

Unless otherwise stated, chemical reagents and solvents were purchased from Sigma-Aldrich, Alfa Aesar, and Fisher Scientific. Anhydrous solvents were obtained from Sigma-Aldrich. Deuterated solvents were purchased from Cambridge Isotope Laboratories.

Unless otherwise indicated, all reactions were conducted at room temperature with stirring under atmospheric pressure. Reactions were monitored with thin layer chromatography (TLC) using aluminum-backed TLC plates silica gel 60 (0.25 mm thickness), viewed under UV light (wavelength 254 nm) or stained with potassium permanganate solution for a non-UV active compound. Silica gel column chromatography was performed on silica gel 60 Å (200–400 mesh) (Sigma-Aldrich).

### Chemical Characterization

Nuclear magnetic resonance (NMR) spectra were recorded in a deuterated solvent, DMSO-*d*_6_, using Bruker ADVANCE III (500 MHz) spectrometers operating at an ambient temperature of 20 °C probe. Data is reported for ^1^H: the chemical shift in ppm (multiplicity, *J* coupling constant in Hz, number of protons) and for ^13^C: the chemical shift in ppm. Multiplicity is presented as follows: s (singlet), d (doublet), t (triplet), q (quartet), m (multiplet), dd (doublet of doublets), dt (doublet of triplets), and td (triplet of doublet).

High-resolution MS (HRMS) was performed using an Agilent QTOF 6545. The specification of the MS instrument is a Jet Stream ESI spray source, an Agilent 1260 Infinity II Quaternary pump HPLC with a 1260 autosampler, a column oven compartment, and a variable wavelength detector. MS was run in either positive or negative ionization mode with the gas temperature at 250 °C. Mass values were stated within the error limits of ±5 ppm mass units.

### Chemical Synthesis and Characterization of Linkers (**1–3**)

#### 3,4-Bis(2-chloroacetamido)benzoic Acid

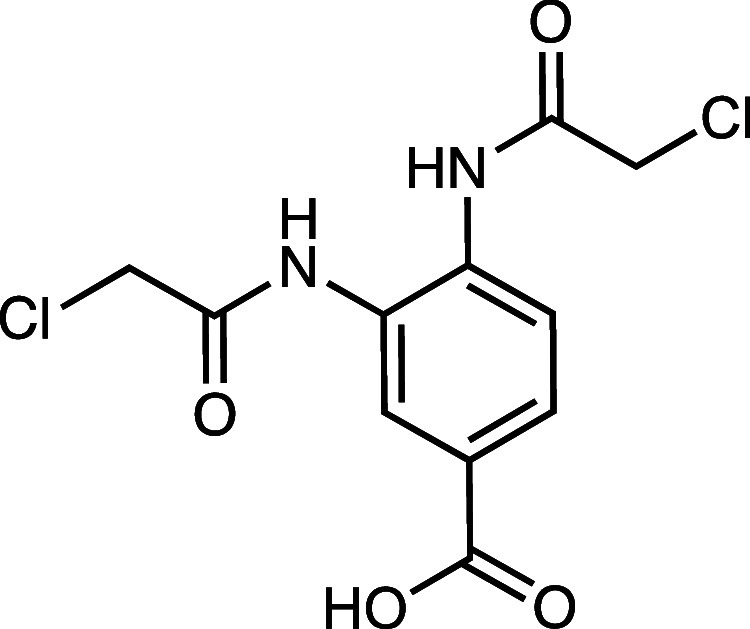



To a stirring cooled solution of 3,4-diaminobenzoic acid (1.0 g, 6.6 mmol) in THF, 2-chloroacetyl chloride (1.62 g, 14.5 mmol, 2.2 equiv) was added dropwise. The mixture was allowed to warm and stirred at room temperature for 2 h. After this, the obtained precipitate was filtered and washed with water 5–6 times and ether. The obtained solid compound was wholly dried under high vacuum to afford 3,4-bis(2-chloroacetamido)benzoic acid as a white solid (1.8 g, 95%).

^1^H NMR (500 MHz, DMSO): δ 9.93 (d, *J* = 3.7 Hz, 2H), 8.15 (s, 1H), 7.85 (d, *J* = 1.1 Hz, 2H), 4.42 (d, *J* = 11.7 Hz, 4H). ^13^C NMR (126 MHz, DMSO): δ 166.49, 165.51, 165.33, 134.55, 129.26, 127.34, 126.77, 126.54, 124.06, 43.32, 43.27. ESI-HRMS: Expected for C_11_H_11_Cl_2_N_2_O_4_ (M + H^+^): *m*/*z* 305.0090. Found: *m*/*z* 305.0095.

#### 2,5-Dioxopyrrolidin-1-yl 3,4-Bis(2-chloroacetamido)benzoate

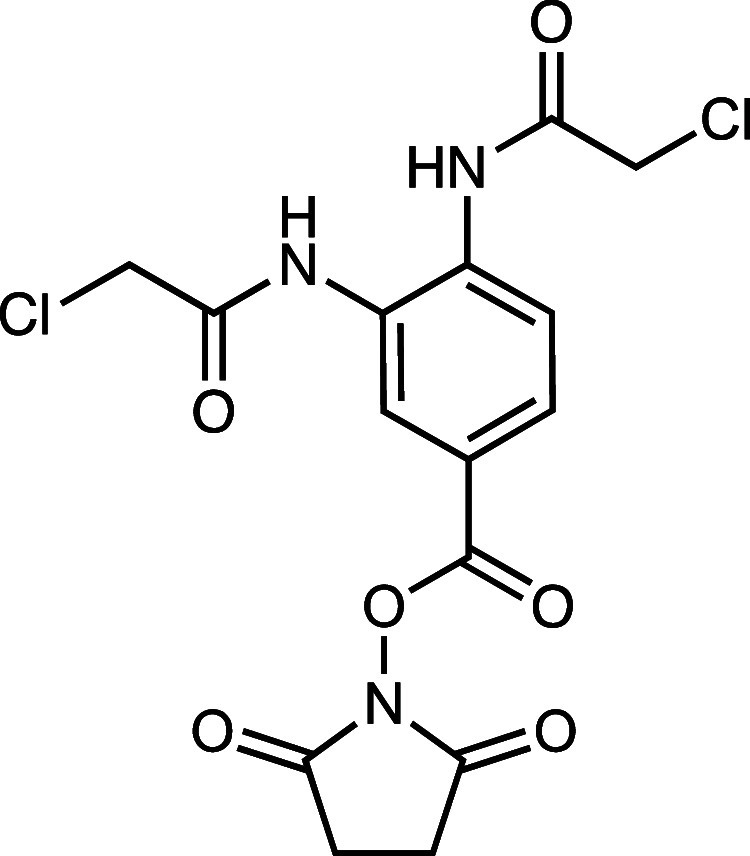



To a stirring solution of 3,4-bis(2-chloroacetamido)benzoic acid (1 g, 3 mmol) and NHS (0.38 g, 3.3 mmol, 1.1 equiv) in THF, EDC·HCl (0.63 g, 3.3 mmol, 1.1 equiv) was added to DMF (5 mL). The reaction mixture was then stirred at room temperature for 2 h; after this, the attained solution was concentrated under reduced pressure. The obtained residue was subjected to a standard workup, and a foamy solid was attained. The activated ester was used as such without further purification.

#### *N*,*N*′-{4-[(2-{2-[2-(2-Azidoethoxy)ethoxy]ethoxy}ethyl)carbamoyl]-1,2-phenylene}bis(2-chloroacetamide)

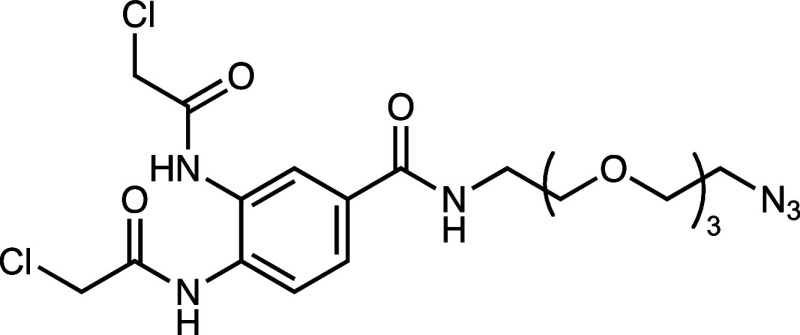



To a stirring anhydrous solution, activated ester (0.50 g, 1.2 mmol) in THF, 2-(2-azidoethoxy)ethan-1-amine (0.34 g, 1.6 mmol, 1.3 equiv) was added. The reaction mixture was then stirred for 1 h at room temperature. After this, the reaction mixture was concentrated under reduced pressure. The obtained residue was subjected to a standard workup. The obtained crude was further purified by silica gel chromatography: (5 to 20% MeOH/DCM) affording *N*,*N*′-{4-[(2-{2-[2-(2-azidoethoxy)ethoxy]ethoxy}ethyl)carbamoyl]1,2-phenylene}bis(2-chloroacetamide) as a white solid (0.3 g, 48%).

^1^H NMR (500 MHz, DMSO): δ 9.87 (d, *J* = 23.9 Hz, 2H), 8.60 (t, *J* = 5.7 Hz, 1H), 8.03 (s, 1H), 7.79 (s, 2H), 4.41 (d, *J* = 9.0 Hz, 4H), 3.64 (dd, *J* = 5.5, 4.3 Hz, 2H), 3.60 (d, *J* = 3.7 Hz, 10H), 3.48 (t, *J* = 5.8 Hz, 4H). ^13^C NMR (126 MHz, DMSO): δ 165.44, 165.24, 133.29, 131.16, 129.19, 125.01, 124.63, 123.85, 69.78, 69.75, 69.66, 69.59, 69.22, 68.84, 49.96, 43.29, 43.23. ESI-HRMS: Expected for C_19_H_26_Cl_2_N_6_O_6_ (M + Na^+^): *m*/*z* 527.1189. Found: 527.1190 *m*/*z*.

#### *N*,*N*′-{4-[(2-{2-[2-(2-Azidoethoxy)ethoxy]ethoxy}ethyl)carbamoyl]-1,2-phenylene}bis(2-iodoacetamide) (Linker **1**)

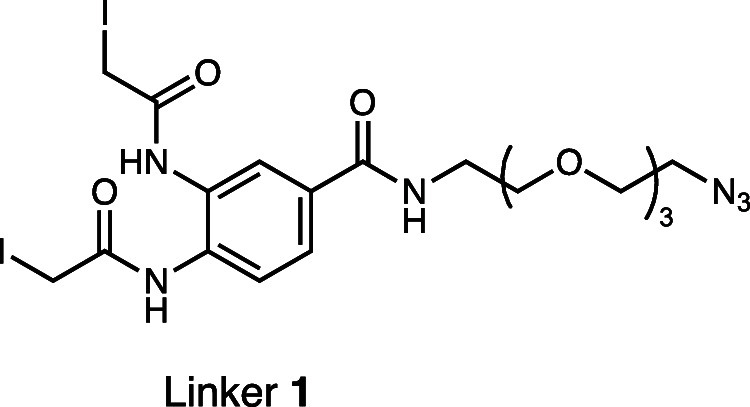



To a solution of *N*,*N*′-{4-[(2-{2-[2-(2-azidoethoxy)ethoxy]ethoxy}ethyl)carbamoyl]-1,2-phenylene}bis(2-chloroacetamide) (1.0 g, 2.0 mmol) in dry acetone (20 mL), KI (1.0 g, 8.0 mmol, 4 equiv) was added. The reaction was refluxed for 3 h. After this, the attained mixture was filtrated, and the solvent was evaporated under reduced pressure. The crude was purified by silica gel chromatography (50 to 70% acetone/chloroform), affording linker **1** as a yellow solid (1.1 g, 81%).

^1^H NMR (500 MHz, DMSO): δ 9.74 (d, *J* = 15.8 Hz, 2H), 8.53 (t, *J* = 5.6 Hz, 1H), 7.97–7.84 (m, 1H), 7.77–7.61 (m, 2H), 3.89 (d, *J* = 10.3 Hz, 4H), 3.54 (dd, *J* = 19.2, 4.4 Hz, 14H). ^13^C NMR (126 MHz, DMSO): δ 167.75, 167.67, 165.85, 134.02, 131.29, 129.81, 125.07, 124.81, 123.86, 70.22, 70.19, 70.12, 70.05, 69.67, 69.33, 50.45, 2.30, 2.20. ESI-HRMS: Expected for C_19_H_26_I_2_N_6_O_6_ (M + Na^+^): *m*/*z* 710.9901. Found: 710.9904 *m*/*z*.

#### Methyl 3,4-Bis(2-bromoacetamido)benzoate (Linker **2**)

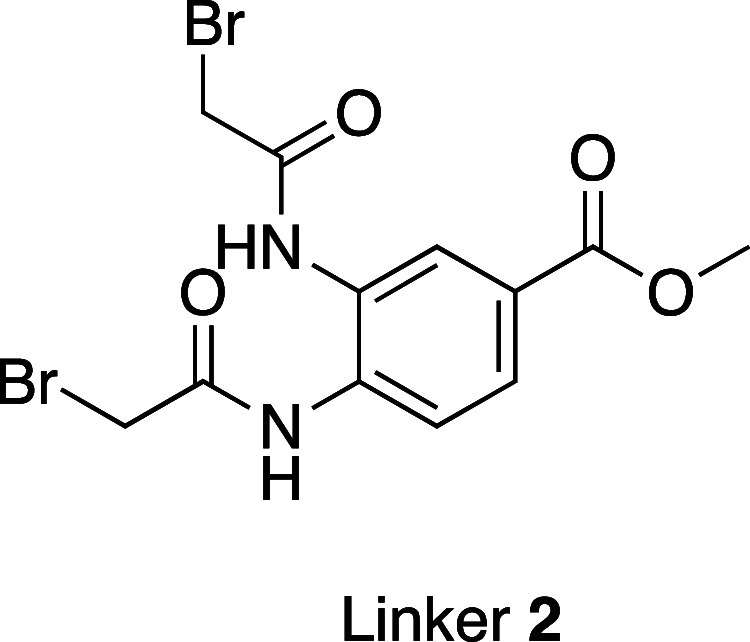



To a stirring cooled solution of methyl 3,4-diaminobenzoate (1.0 g, 6.0 mmol) in DCM and TEA (1.35 g, 13.3 mmol, 2.2 equiv) was added dropwise 2-bromoacetyl bromide (2.65 g, 13.2 mmol, 2.2 equiv) over 30 min. The obtained precipitate was filtered and washed with water, followed by washing with ether. The attained precipitate was dried under high vacuum to afford the linker **3** as a white solid (1.90 g, 78%).

^1^H NMR (500 MHz, DMSO-*d*_6_): δ 9.98 (d, *J* = 5.7 Hz, 2H), 8.16 (s, 1H), 7.88 (s, 2H), 4.21 (d, *J* = 13.0 Hz, 4H), 3.92 (s, 3H). ^13^C NMR (126 MHz, DMSO-*d*_6_): δ 165.60, 165.44, 135.02, 129.36, 126.60, 126.17, 126.00, 123.99, 52.22, 30.17. ESI-HRMS: Expected C_12_H_12_Br_2_N_2_O_4_ (M + 1^+^): 406.9242 *m*/*z*. Found: *m*/*z* 406.9241.

#### 3,5-Bis(2-chloroacetamido)benzoic Acid

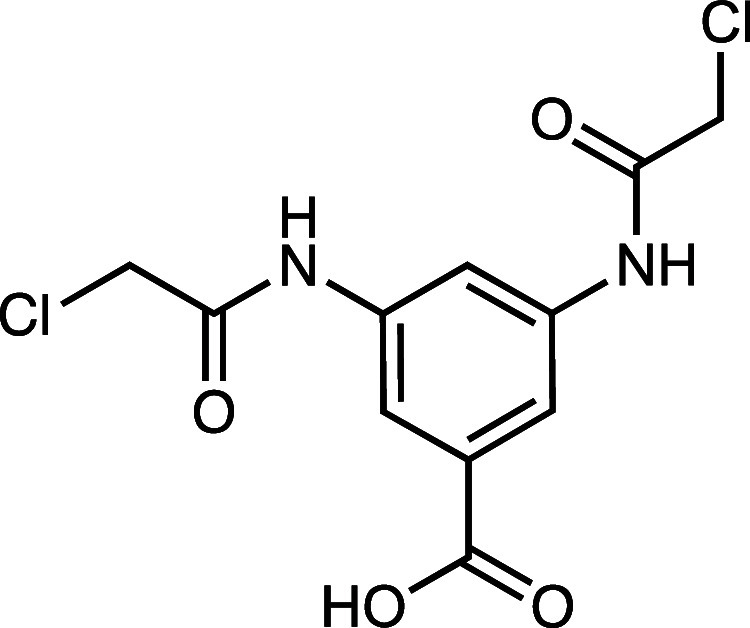



A similar synthesis procedure was adopted for the synthesis of 3,5-bis(2-chloroacetamido)benzoic acid, as described for 3,4-bis(2-chloroacetamido)benzoic acid (yield 94%).

^1^H NMR (500 MHz, DMSO): δ 10.59 (s, 2H), 8.19 (s, 1H), 7.96 (d, *J* = 2.0 Hz, 2H), 4.28 (s, 4H).

^13^C NMR (126 MHz, DMSO): δ 167.23, 165.44, 139.55, 132.30, 115.96, 114.40, 44.01. ESI-HRMS: Expected for C_11_H_11_Cl_2_N_2_O_4_ (M + H^+^): *m*/*z* 305.0090. Found: 305.0094 *m*/*z*.

#### 2,5-Dioxopyrrolidin-1-yl 3,5-Bis(2-chloroacetamido)benzoate

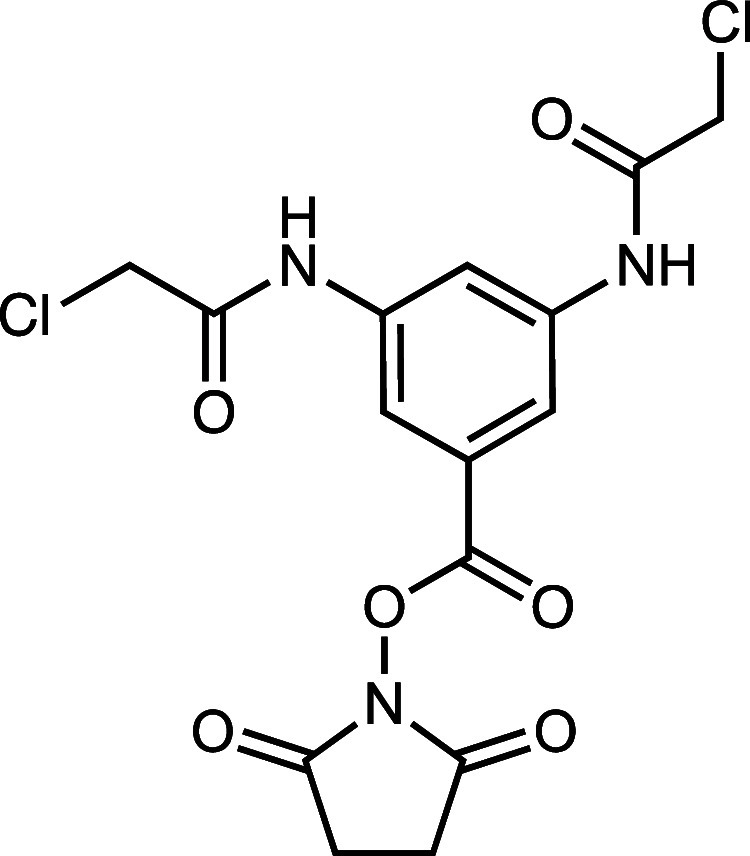



A similar synthesis procedure was adopted for the synthesis of 2,5-dioxopyrrolidin-1-yl 3,5-bis(2-chloroacetamido)benzoate, as described for 2,5-dioxopyrrolidin-1-yl 3,4-bis(2-chloroacetamido)benzoate.

#### *N*,*N*′-{4-[(2-{2-[2-(2-Azidoethoxy)ethoxy]ethoxy}ethyl)carbamoyl]-1,3-phenylene}bis(2-chloroacetamide)

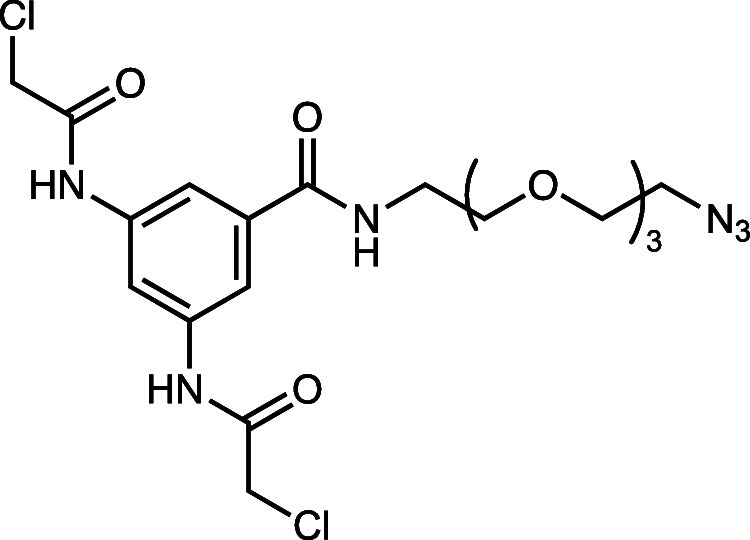



A similar synthesis procedure was adopted for the synthesis of 2 *N*,*N*′-{4-[(2-{2-[2-(2-azidoethoxy)ethoxy]ethoxy}ethyl)carbamoyl]-1,3-phenylene}bis(2-chloroacetamide), as described for *N*,*N*′-{4-[(2-{2-[2-(2-azidoethoxy)ethoxy]ethoxy}ethyl)carbamoyl]-1,2-phenylene}bis(2-chloroacetamide) (yield 49%).

^1^H NMR (500 MHz, DMSO): δ 10.56 (s, 2H), 8.54 (t, *J* = 5.6 Hz, 1H), 8.14 (t, *J* = 2.0 Hz, 1H), 7.79 (d, *J* = 1.9 Hz, 2H), 4.32 (s, 4H), 3.61 (dd, *J* = 17.2, 4.7 Hz, 12H), 3.47 (d, *J* = 6.3 Hz, 4H).

^13^C NMR (126 MHz, DMSO): δ 166.69, 165.35, 139.22, 136.61, 114.41, 113.34, 79.62, 70.27, 70.24, 70.14, 70.03, 69.69, 69.26, 50.44, 43.99. ESI-HRMS: Expected for C_19_H_26_Cl_2_N_6_O_6_ (M + Na^+^): *m*/*z* 527.1189. Found: 527.1181 *m*/*z*.

#### *N*,*N*′-{4-[(2-{2-[2-(2-Azidoethoxy)ethoxy]ethoxy}ethyl)carbamoyl]-1,3-phenylene}bis(2-iodoacetamide) (Linker **3**)

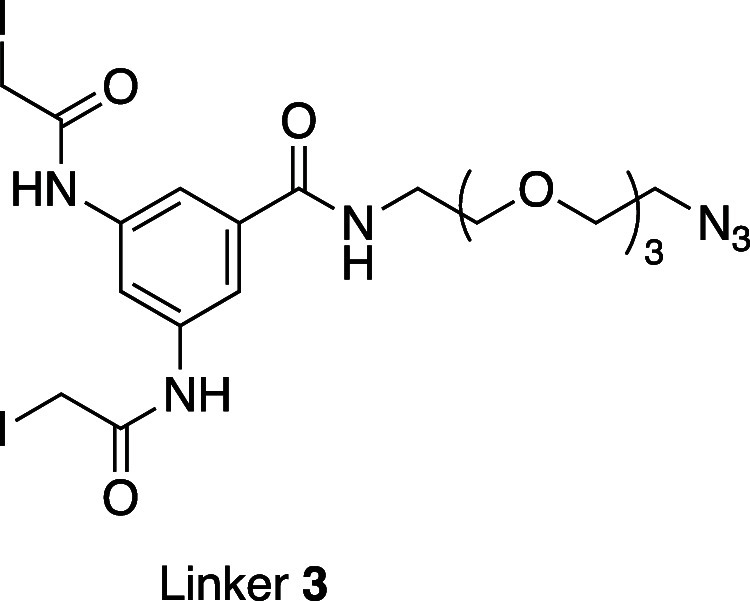



A similar synthesis procedure was adopted for the synthesis of linker **2**, as described above for linker **1** (yield 83%).

^1^H NMR (500 MHz, DMSO): δ 10.50 (s, 2H), 8.47 (t, *J* = 5.7 Hz, 1H), 8.04 (t, *J* = 2.0 Hz, 1H), 7.68 (d, *J* = 2.0 Hz, 2H), 3.84 (s, 4H), 3.58 (dd, *J* = 5.6, 4.3 Hz, 2H), 3.57–3.49 (m, 10H), 3.39 (dt, *J* = 8.4, 5.2 Hz, 4H). ^13^C NMR (126 MHz, DMSO): δ 164.94, 164.45, 137.23, 134.31, 111.45, 110.27, 67.90, 67.87, 67.77, 67.66, 67.33, 66.89, 48.07, −0.50.

Expected for C_19_H_26_I_2_N_6_O_6_ (M + Na^+^): *m*/*z* 710.9901. Found: 710.9894 *m*/*z*.

### Bioconjugation Chemistry General Notes

Amab (Tecentriq) 1200 mg samples were generously provided as gifts from Pharmaxo Scientific. Amicon Ultra (10 kDa) centrifugal filters were purchased from Sigma-Aldrich and used for buffer exchange.

SDS-PAGE was used to separate protein samples by using the Invitrogen Mini Gel system. Novex gel cassettes (1.0 mm, Invitrogen) were used to prepare 4% stacking gel and 10% acrylamide gel (nonreducing glycine gel). Prestained Ladder (PageRuler, Thermo Fisher) was used to estimate the proteins’ size (MW).

Protein samples were prepared for liquid chromatography–MS (LC–MS) by buffer exchanging into deionized water using Amicon Ultra spin filters. Samples were prepared to 2 mg/mL where possible. LC–MS analysis was performed using an Agilent Electrospray Quadrupole Time-of-Flight (ESIQTOF) 6545 coupled to an Agilent 1260 Infinity II Quaternary pump HPLC. Data analysis was performed using MassHunter BioConfirm 10.0.

### Procedure for Reduction of Antibodies

Before the conjugation reaction was started, the antibodies were exchanged in the conjugation buffer (0.1 M Tris, 0.15 M NaCl, 5 mM EDTA, pH 7.5). A fresh solution of TCEP was also prepared in the same conjugation buffer. 1.1, 2.2, or 5 equiv of TCEP was added to the antibody and incubated at room temperature for 2 h.

### Conjugation Chemistry to Attain Amab Conjugates

To prepare *Amab conjugate* 1, TCEP (1.1 equiv) was used to reduce Amab (5 mg/mL) for 2 h at room temperature. Then, linker **1** (2 equiv) was added to the partially reduced antibody and allowed to react overnight at room temperature.

To attain *Amab conjugate 2*, TCEP (2.2 equiv) was used to reduce Amab (5 mg/mL) for 2 h at room temperature. Then, 3 equiv of the linker **2** was added to the partially reduced antibody and left to react at room temperature overnight. The attained Amab conjugate was purified via diafiltration into a conjugation buffer to remove unreacted linkers. The second reduction step to attain linking at the hinge region was performed by reduction with TCEP (2.2 equiv) for 2 h, followed by overnight incubation with linker **3** (5 equiv).

To obtain *Amab conjugate* 3, Amab (5 mg/mL) was reduced with TCEP (5 equiv) for 2 h at room temperature. Then, 20 equiv of Maleimide-PEG3-N3 (purchased from Thermo Fisher Scientific) was added to the fully reduced antibody and allowed to react at room temperature overnight.

*Amab conjugates* were purified through diafiltration into PBS buffer to remove unreacted linkers and stored in the fridge. The products were resolved by using SDS-PAGE.

### Nanoformulation

Eudragit L-100 (EL100) NPs were prepared using a previously published nanoprecipitation protocol with some amendments.^44^ 10 mg of Eudragit L-100 was dissolved in 10 mL of ethanol; after that, the polymer solution was added to 10 mL of deionized water (0.6 mL per min) and the NPs formed immediately; coumarin 6- and PTX-loaded NPs were prepared following the same protocol by dissolving coumarin 6 or PTX in ethanol with the polymer, respectively.

### Surface Functionalization of NPs

Surface functionalization was achieved over two steps: the activation and dialysis steps before being functionalized with antibodies.

Activation was performed by incubation with NHS/EDC·HCl (10 equiv relative to polymer concentration) for 2 h affording NP-NHS followed by overnight dialysis. Surface functionalization with unmodified Amab was achieved by adding 3 nmol of Amab to 1 mg of NP-NHS suspended in PBS buffer.

To introduce a clickable moiety, dibenzocyclooctyne-amine (10 mol equiv relative to polymer concentration, purchased from Sigma-Aldrich) was added to NP-NHS for 2 h, followed by an overnight dialysis step. When required, 3 nmoles of *Amab conjugate 1*, *Amab conjugate 2*, or *Amab conjugate 3* was incubated with 1 mg of cyclooctyne-activated polymer at room temperature. After gentle stirring for 6 h, the NPs were subjected to dialysis in PBS.

### Characterization of the NP

After diluting the samples with deionized water and monitoring the light scattering at a 173° angle to the incident radiation at 25 °C, the mean NP hydrodynamic diameters were determined using a Malvern (TM) Zetasizer outfitted with a 10 mW He–Ne laser with a 633 nm wavelength. Using the same Malvern (TM) Zetasizer, the NPs’ zeta potential in water was measured.

The coupling % of Amab was calculated according to^45^ Liang et al. using the following equation using a plate reader (Thermo Scientific Multiskan Sky, USA)—the concentration of Amab prior to and after the surface fabrication in the eluant.
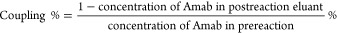


The percentage of the entrapped drug was determined by assessing the amount of the drug entrapped in the NPs following 24 h of dialysis against 1 L of deionized water, where a certain amount of freeze-dried NPs were reconstituted in ethanol and analyzed using a previously validated UV–vis spectrophotometric method at 266 nm (Figure S3). The PTX-entrapped percentage was calculated using the following equation
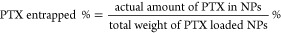


### Cellular Uptake and Cytotoxicity Study

#### Cell Culture

The EMT6 (CRL-2755) cell line was obtained from the American Type Culture Collection (ATCC) (Manassas, VA, USA) and stored in liquid nitrogen. Cells were passaged twice weekly upon reaching 70–80% confluency, and low passage numbers (≤12) were used in all experiments. EMT6 cells were subcultured in DMEM-high glucose medium 1× (DMEM-HG, Euroclone, Italy) supplemented with 10% (v/v) heat-inactivated fetal bovine serum (Biowest, France), 1% penicillin–streptomycin solution 100× (Euroclone, Italy), and 1% l-glutamine 100× (Euroclone, Italy). The cells were maintained at 37 °C in a humidified 5% CO2 incubator (Esco, Singapore).

#### Flow Cytometry

To quantify the percentage of the cellular uptake of fluorescent NPs by the EMT6 cell line, cells were plated in 12-well plates (SPL, Korea) at a seeding density of 1 × 10^5^ cells/well for 24 h. Next, cells were divided into two groups: blocking and no blocking. For the blocking group, all cells were incubated with unmodified Amab (20 μg/mL) for 15 min at 4 °C. Then, cells were washed with PBS.

Following that, both blocked and unblocked groups were incubated with a final concentration of 125 μg polymer/mL from different treatment groups by taking 25% of 200 μL of media for 45 min at 37 °C. Untreated cells were used as a negative control. Then, all treatments were washed out, and cells were incubated with a cell culture medium for 45 min. Following that, all cells (blocked and unblocked) were trypsinized by trypsin 1× (Euroclone, Italy) and collected and then acquired by flow cytometry Canto2 (BD, Biosciences, FACS DIVA version8). Data analysis and interpretation were done using FlowLogic software, version 3.

#### Confocal Microscopy

For confocal microscopy, EMT6 cells were plated on coverslips placed in 12-well plates at a seeding density of 20 × 10^3^ cells/coverslip. Next, cells were divided into two groups: with and without blocking. For the blocking group, cells were incubated with unmodified Amab (20 μg/mL) for 15 min at 4 °C. Then, cells were washed with PBS prior to incubation with 125 μg polymer/mL from different treatment groups. Untreated cells were used as a negative control. Then, treatments were washed out, and cells were incubated with cell culture media for 1 h. Then, all treated cells (blocked and unblocked) were washed twice with PBS and fixed by 4% PFA for 10 min. Then, fixed cells were washed thrice with the washing buffer. After that, 4′,6-diamidino-2-phenylindole (DAPI) stain (Thermo Fisher, Waltham, MA, USA) was added to the cells and incubated for 5 min, followed by a washing step with PBS. Finally, coverslips were transferred into glass slides loaded with one drop of mounting medium (DAKO, Glostrup, Denmark). At last, confocal images were acquired via a laser scanning microscope 780 (Zeiss, Oberkochen, Germany). The objective used for acquiring the images was a Plan-Apochromat 63X/1.4 Oil DIC M27. Lasers of 405 and 457 nm were activated for excitation of the nuclear stain DAPI and coumarin 6, respectively. Detector ranges for emission signals were 410–556 nm for DAPI and 501 for coumarin 6.

#### MTT Assay

For the MTT assay, EMT6 cells were cultured at a seeding density of 5000 cells/well in 96 tissue culture well plates (SPL, Korea) for 24 h. Next, cells were treated with either unloaded NPs with the following concentrations: 125, 100, 75, 50, 25, and 12.5 μg polymer/mL or the treatment groups (NPs loaded with PTX) for 45 min at 37 °C. Then, all treatments were washed out, and all cells were incubated with a cell culture medium for the next 24 h. Unloaded NPs were used as a negative control, whereas free PTX (with equivalent concentrations) was used as a positive control.

Following 24 h, an MTT assay was performed by adding 10 μL of MTT salt into each well for 3 h. Then, 100 μL of stop solution was used to stop the reaction. The absorbance was measured at 570 nm (GloMax, Promega, USA). The values of 50% inhibitory concentration (IC_50_) were calculated using GraphPad Prism version 7.01.

#### Apoptosis/Necrosis Assay

To determine the cytotoxic effect of PTX NPs conjugated with Amab, cells were plated in 12-well plates (SPL, Korea) at a seeding density of 1 × 10^5^ cells/well for 24 h. Next, cells were divided into two groups: with blocking and without blocking. EMT6 cells were blocked when required by incubating them with Amab (20 μg/mL) for 15 min at 4 °C. Then, cells were washed with PBS. After that, cells were incubated with treatment groups NPs (PTX) for 45 min at 37 °C. EMT6 cells treated with PTX (32 μg/mL, equivalent to the PTX NPs) were used as a positive control. Untreated cells were used as a negative control. Then, treatments were washed out, and cells were incubated with cell culture media for 24 h. Following that, all cells were trypsinized by trypsin 1× (Euroclone, Italy) and collected; then, cells were stained by an Annexin V PI kit (eBioscience, USA). All samples were prepared according to the manufacturer’s instructions. Finally, stained samples were acquired by Canto2 flow cytometry (BD, Biosciences, FACSDiva version 8). Data analysis and interpretation were performed using FlowLogic software, version 3.

#### Statistical Analysis

If otherwise stated, data are presented as the mean + SD (*n* = number of experiments). Data were considered significant when the *p* value ≤ 0.05, where *, **, ***, and **** represent *p* values < 0.05, <0.01, <0.001, and <0.0001, respectively. Statistics were calculated using GraphPad Prism version 7.01.

## Supporting Information Available

Mechanism of action of NPs fabricated with anti-PDL1 antibodies; conventional conjugation chemistries employed to develop ADCs; calibration curve of PTX; coupling % of the nanoformulations; statistical analysis of cellular uptake and cell viability; characterization of Amab conjugates using protein MS; and NMR characterization of the linkers (PDF)
