# Supplementary material for: Facile Rebridging Conjugation Approach to Attain Monoclonal Antibody-Targeted Nanoparticles with Enhanced Antigen Binding and Payload Delivery
Source: Bioconjug Chem. 2024 Sep 10;35(10):1491–502. doi: 10.1021/acs.bioconjchem.4c00275 (PMC11487529; doi:10.1021/acs.bioconjchem.4c00275)
Supplement: Supplementary file 1 — bc4c00275_si_001.pdf [file bc4c00275_si_001.pdf]

Supporting Information

A facile rebridging conjugation approach to attain monoclonal antibodies-targeted nanoparticles with enhanced antigen binding and payload delivery

Bayan Alkhawaja[1*], Duaa Abuarqoub[1,2], Mohammad Al-natour[1], Walhan Alshaer[2], Qasem Abdallah[1], Ezaldeen Esawi[2] , Malak Jaber[1], Nour Alkhawaja[1], Bayan Y. Ghanim[4], Nidal Qinna[4], Andrew G. Watts[3*]

[1] Faculty of Pharmacy and Medical Sciences, The University of Petra, Amman 11196, Jordan

[2] Cell Therapy Center, University of Jordan, Amman 11942, Jordan

[3] Department of Life Sciences, University of Bath, Claverton Down, BA2 7AY, Bath, UK

[4] University of Petra Pharmaceutical Center, Faculty of Pharmacy and Medical Sciences, Petra University, Amman 11196, Jordan

Table of Contents

1- Supplementary Figures 2

2- Characterisation of Amab conjugates using protein MS. 5

3- Supplementary tables 3

4- ^{1}H and ^{13}C NMRs Characterisation of the linkers (1-3) 10

References 27

1- Supplementary figures

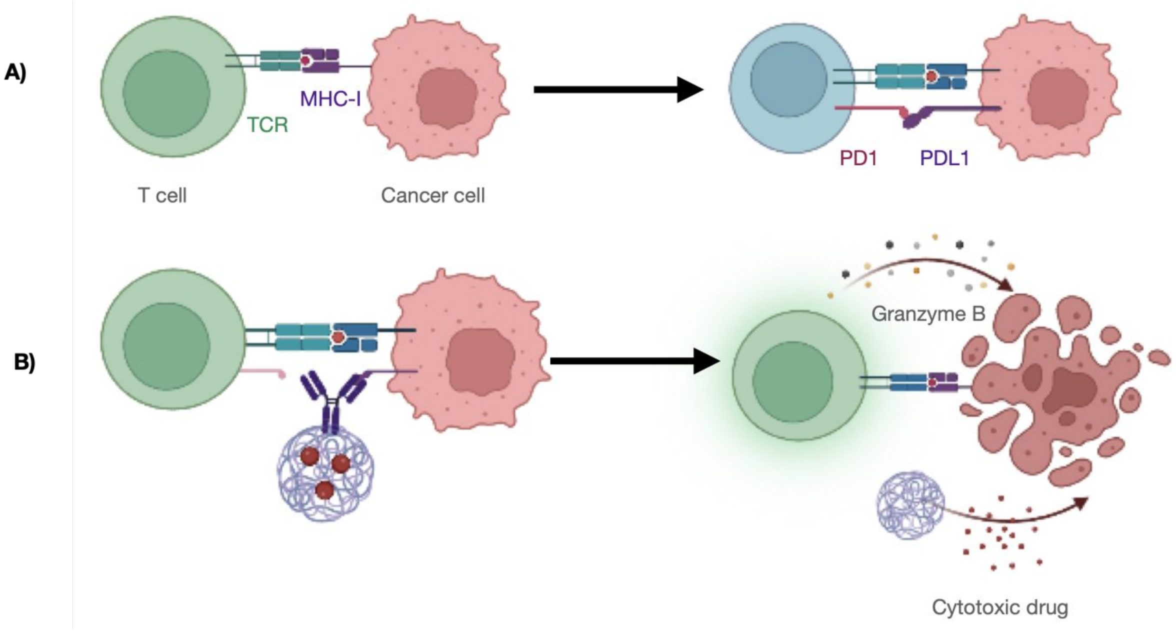

Figure S1. Mechanism of action of NP fabricated with anti-PDL1 antibodies. A) The CD8+ T cell activates upon recognising the tumour antigen presented on MHC class I and consequently induces the expression of PDL1 on tumour cells. PDL1 conjugates the elevated PD1 on T cell surface, triggering the inhibitory effect of the PD1/PDL1 axis.[1] B) Anti-PDL1 antibody linked with NP-carrying cytotoxic drug blocks the interaction of PD1 and PDL1 and abolishes the inhibition of CD8+ T cell, thus enhancing the antitumor activity. Created with BioRender.com

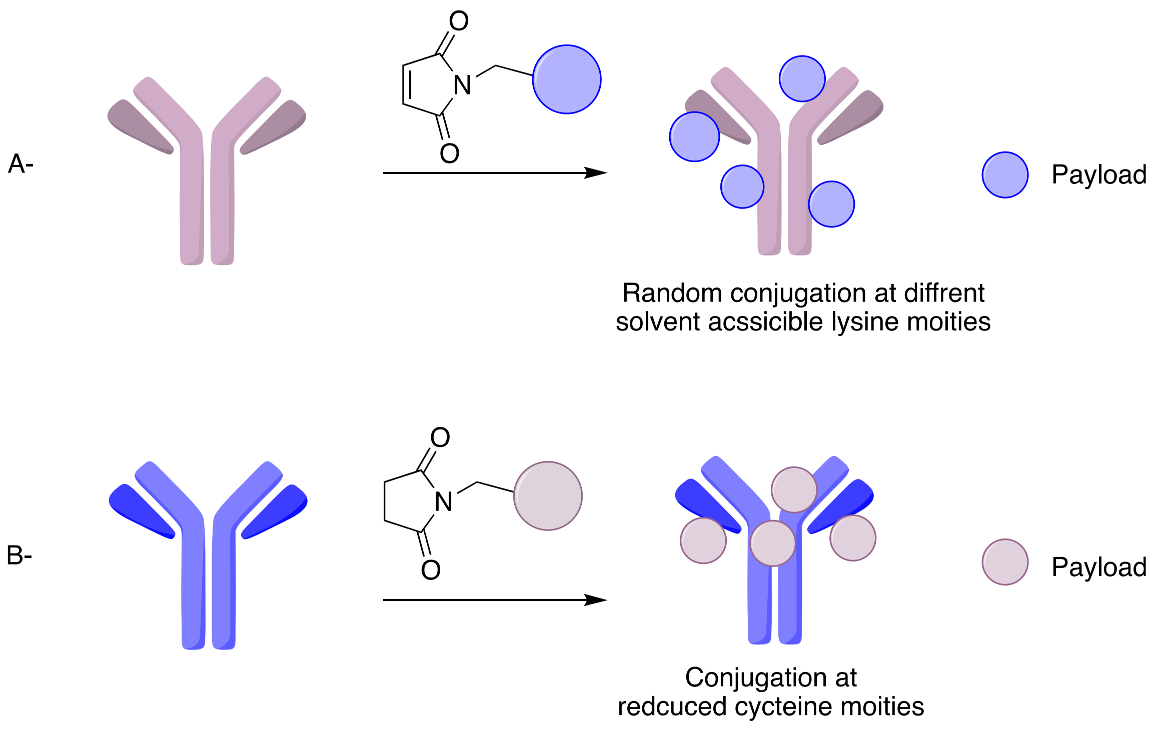

Figure S2. Conventional conjugation chemistries are employed to develop ADCs.

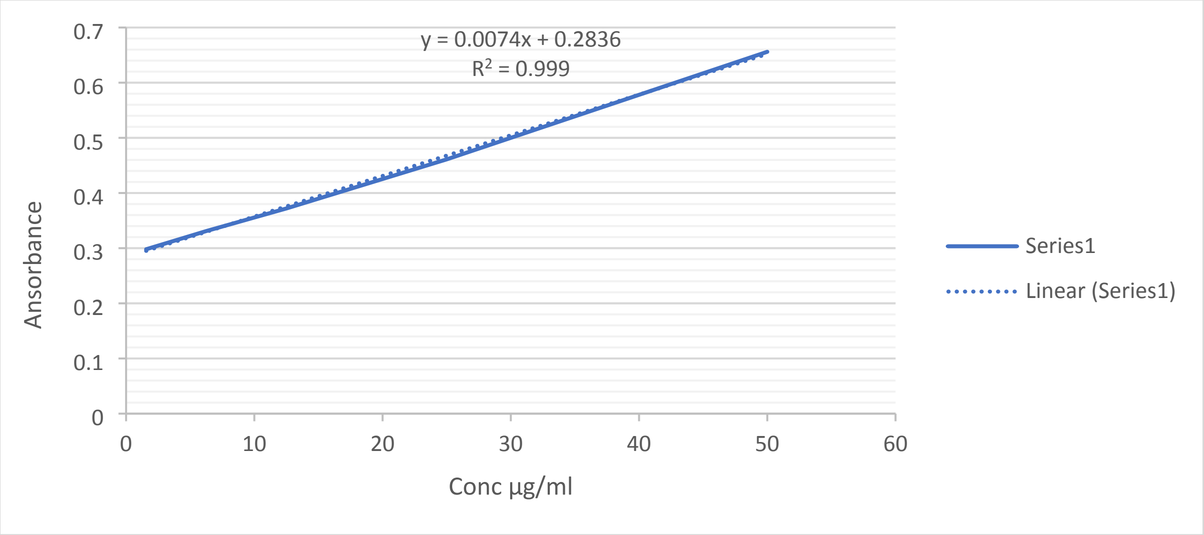

Figure S3. Calibration curve of PTX.

2- Supplementary Tables

Table S1. Coupling % of the nanoformulations

Nanoformulations	Coupling (%)
NP-NHS Ab	86%
NP-Mal Ab	70%
NP-Fab BisHalide Ab	74%
NP6-Fc BisHalide Ab	73%

Table S2. Statistical analysis -cellular uptake

Dunnett's multiple comparisons test	*p-value*
NP5 vs. NP2	****
NP5 vs. NP4	****
NP5 vs. NP7	****
NP5 vs. NP5- With blocking	****
NP5 vs. NP6	*
NP6 vs. NP2	****
NP6 vs. NP4	****
NP6 vs. NP7	***
NP6 vs. NP6- With blocking	ns

Table S3. Statistical analysis of cell viability

Dunnett's multiple comparisons test	*p*-value
PTX vs. NP7	****
PTX vs. NP5	****
PTX vs. NP6	****
PTX vs. NP4	****
PTX vs. NP2	****
NP2 vs. NP7	****
NP2 vs. NP5	***
NP2 vs. NP6	**
NP2 vs. NP4	****
NP7 vs. NP5	****
NP7 vs. NP4	****
NP7 vs. NP6	****

3- Characterisation of Amab conjugates using protein MS.

1- *Amab*

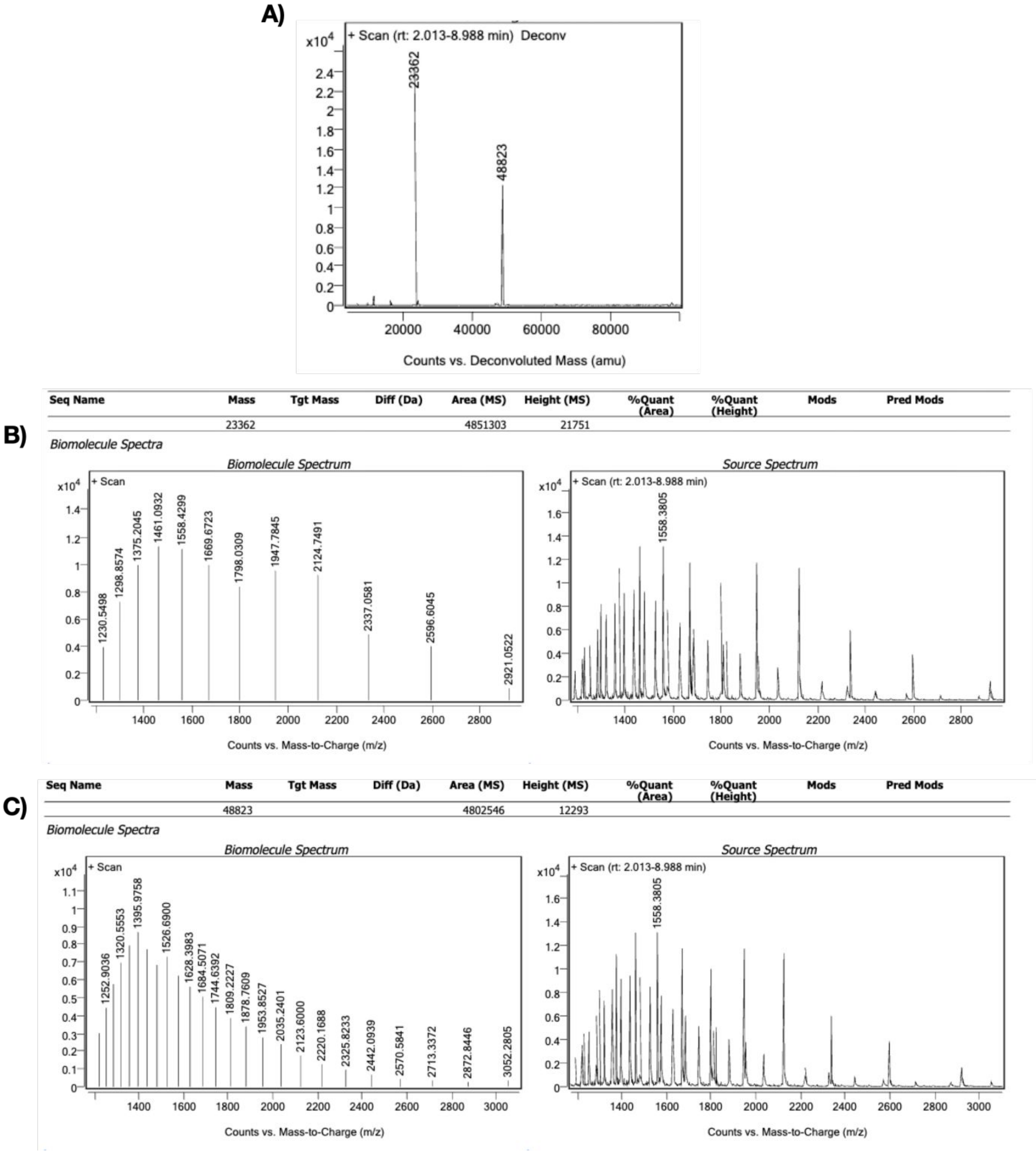

Figure S4. A- Deconvoluted spectrum protein MS of *Amab*, showing main peaks at 23,362 Da, 48,823 Da and 72,616 Da. B & C - Non-deconvoluted spectrum protein MS of *Amab* major products.

2- *Amab conjugate 1* (Amab-Fab-N3)

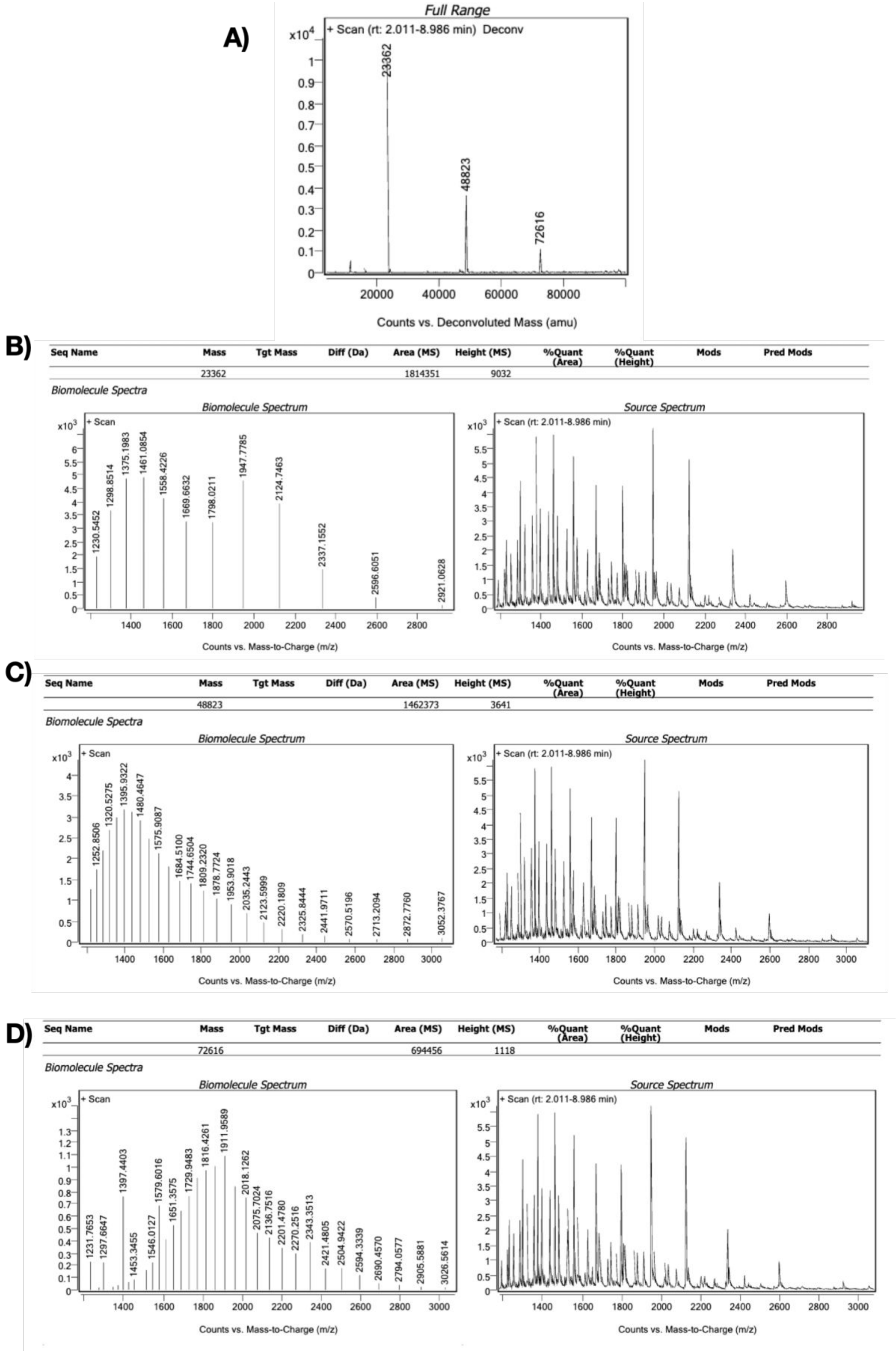

Figure S5. A- Deconvoluted spectrum protein MS of *Amab conjugate 1*, showing main peaks at 23,362 Da, 48,823 Da and 72,616 Da. B, C &D- Non-deconvoluted spectrum protein MS of *Amab conjugate 1* significant product.

3- *Amab conjugate 2* (Amab-Fc-N_3)

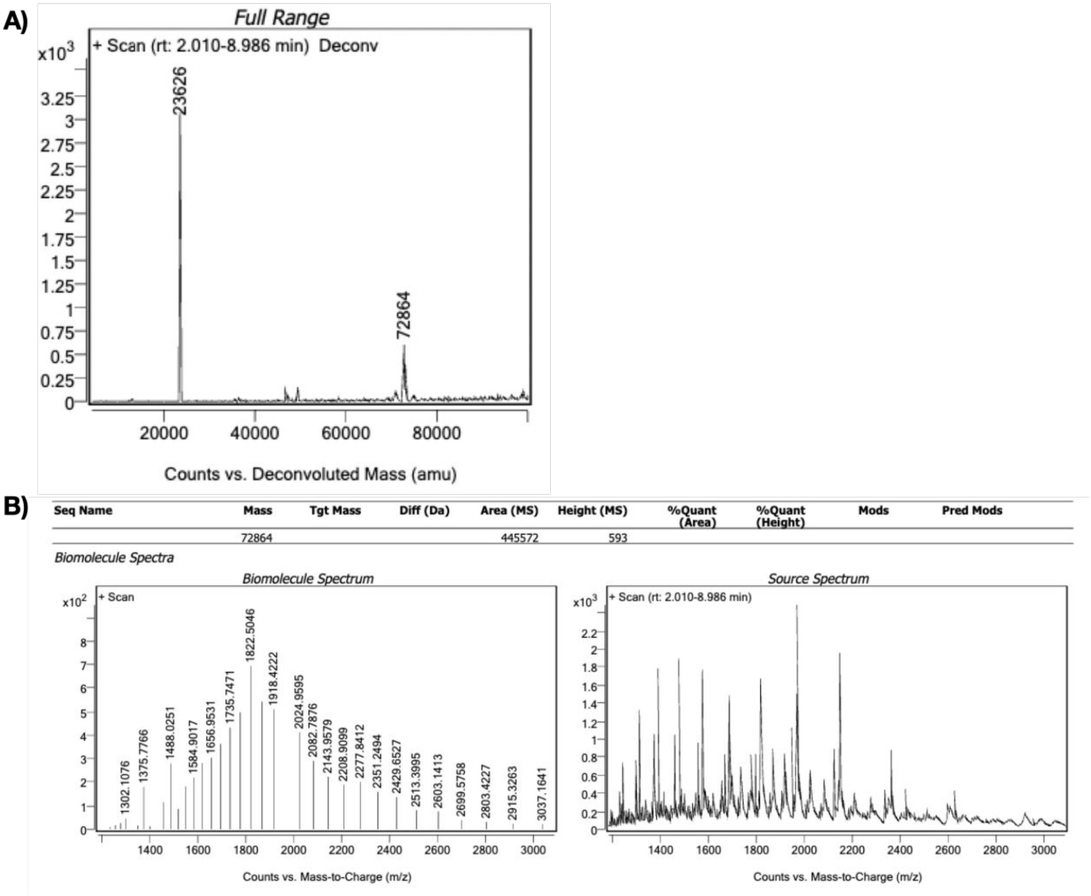

Figure S6. A- Deconvoluted spectrum protein MS of *Amab conjugate 2*, showing major peaks at 72,864 Da. B- Non-deconvoluted spectrum protein MS of *Amab conjugate 2*.

4- *Amab conjugate 3* (Amab-Mal)

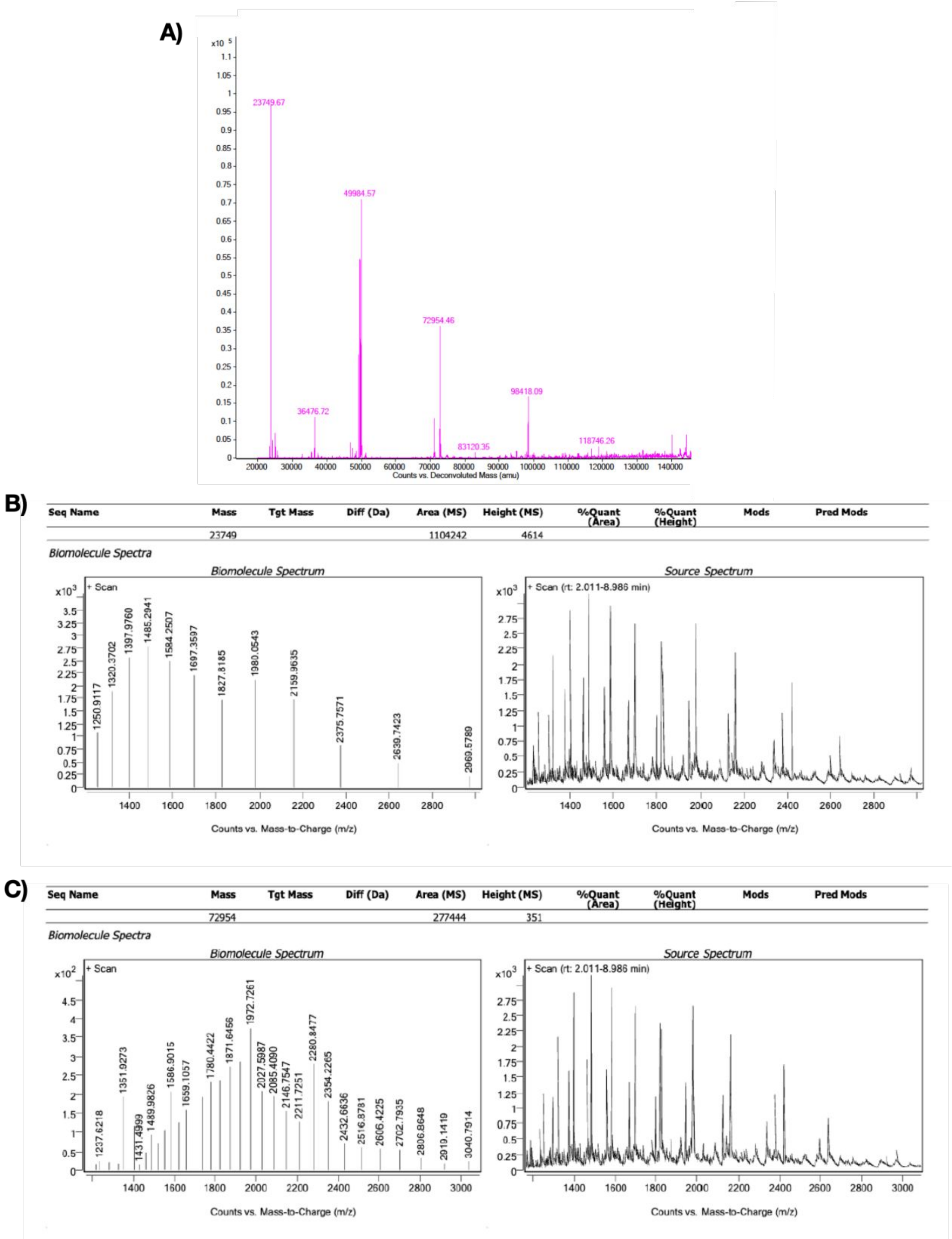

Figure S7. A- Deconvoluted spectrum protein MS of *Amab conjugate 3*, showing main peaks at 23,749 Da and 49,984 Da. B- Non-deconvoluted spectrum protein MS of *Amab conjugate 3.*

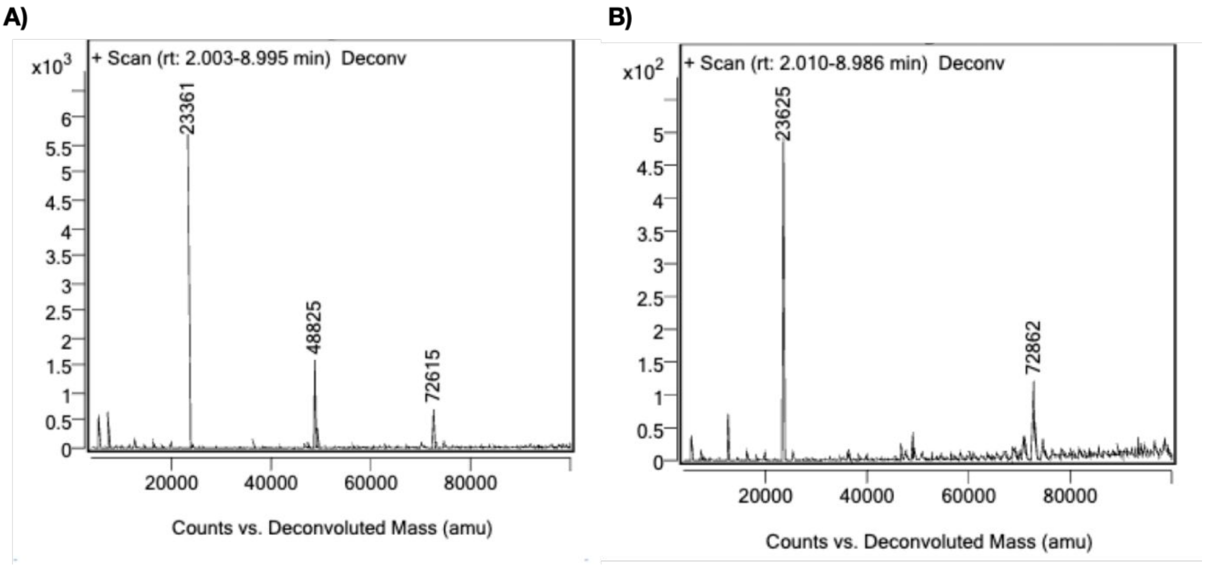

Figure S8. A- Deconvoluted spectrum protein MS of A) *Amab conjugate 1 and B) Amab conjugate 2 after being* stored in the refrigerator for up to 3 months.

4- ^{1}H and ^{13}C NMRs Characterisation of the linkers (1-3)

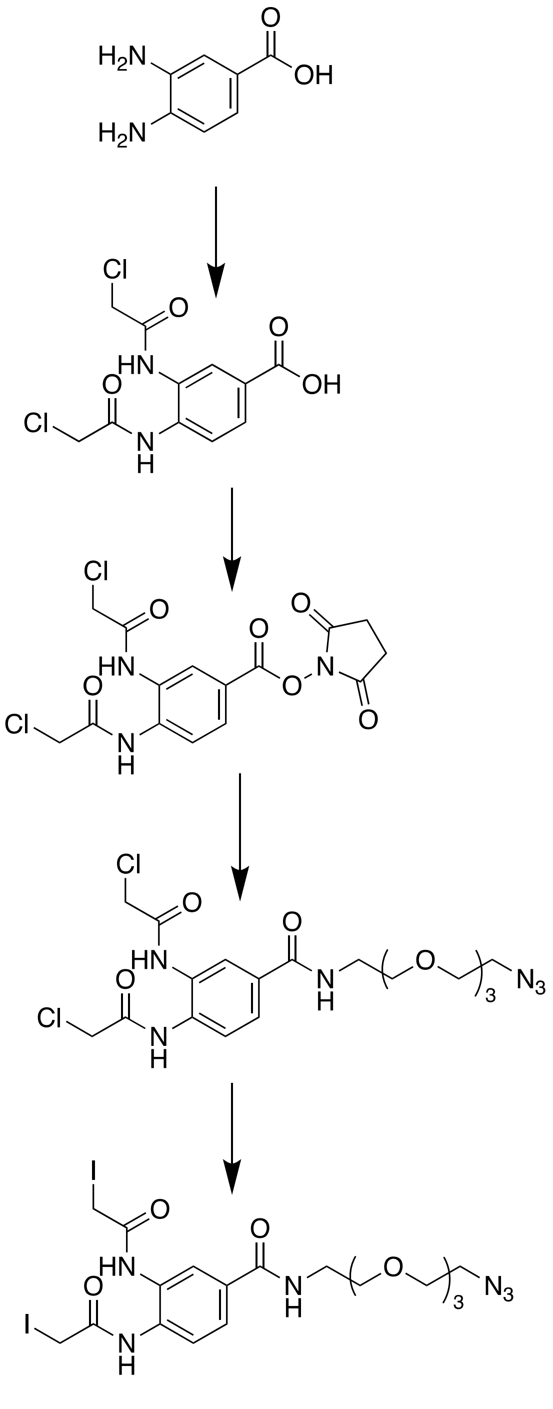

Linker **1**

Scheme S1. The chemical synthesis of Linker **1.**

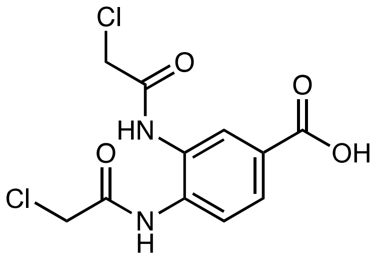

3,4-bis(2-chloroacetamido)benzoic acid

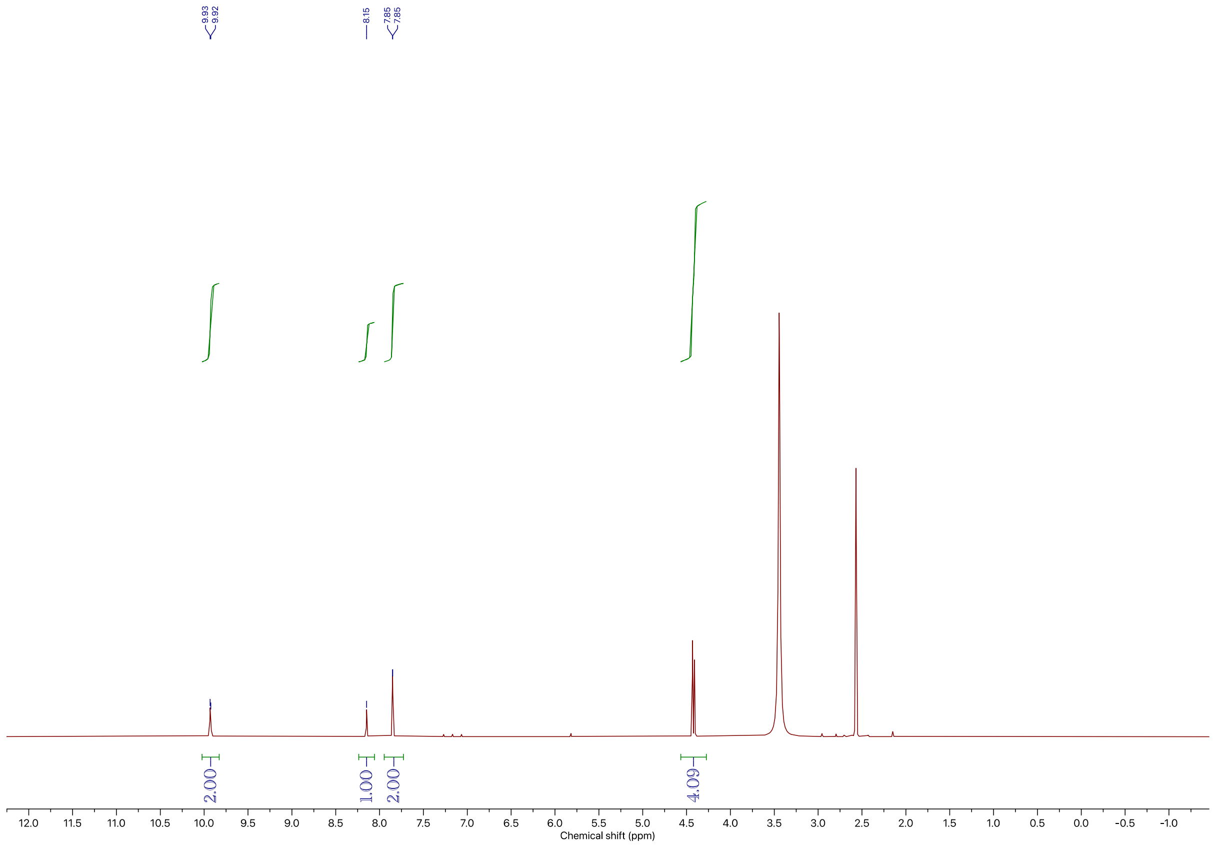

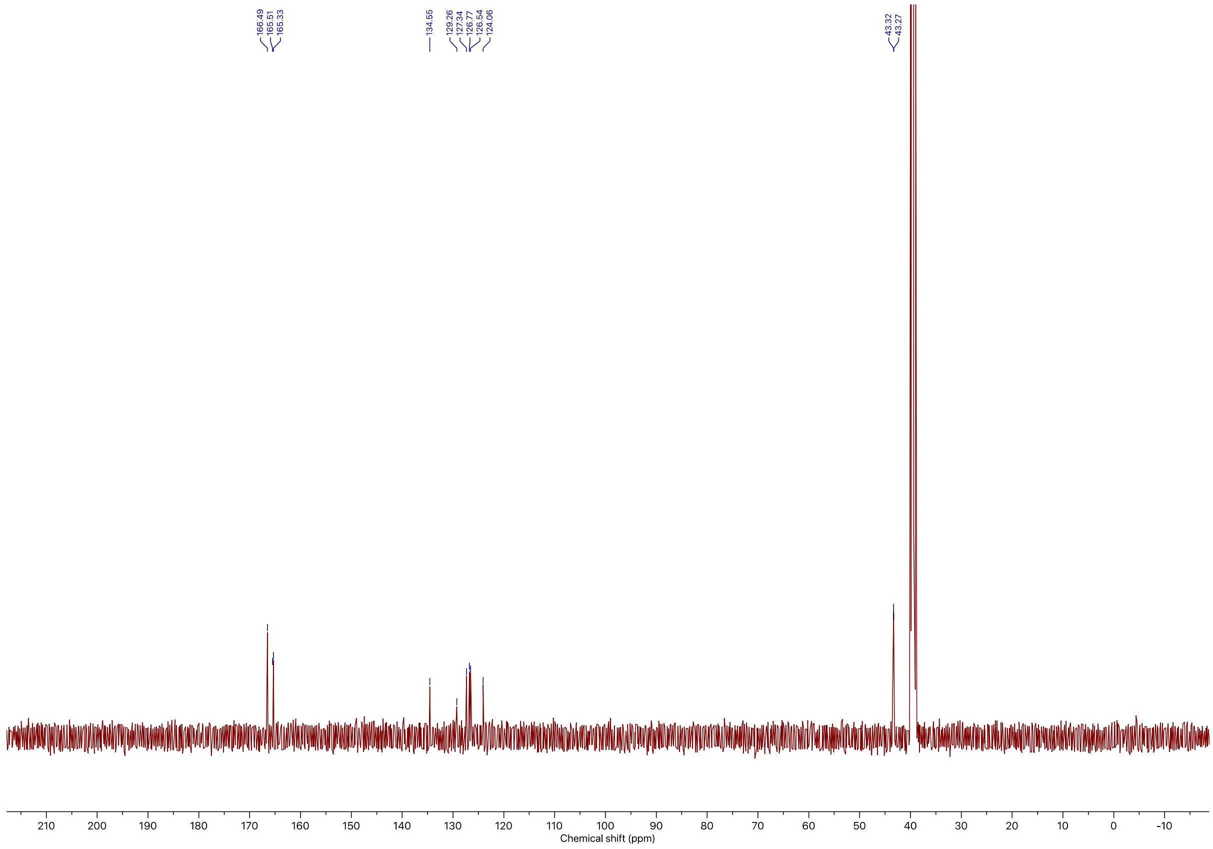
166.49
165.51
165.33
134.55
129.26
127.34
126.77
126.54
124.06
43.32
43.27
210
200
190
180
170
160
150
140
130
120
110
100
90
80
70
60
50
40
30
20
10
0
-10
Chemical shift (ppm)

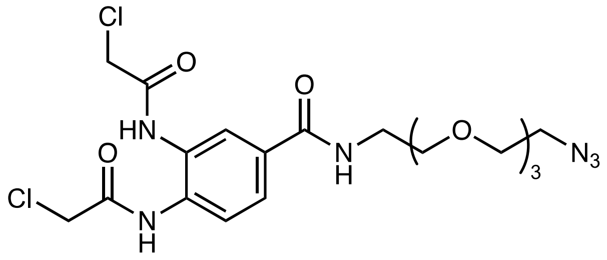

N,*N*'-(4-((2-(2-(2-(2-azidoethoxy)ethoxy)ethoxy)ethyl)carbamoyl)-1,2-phenylene)bis(2-chloroacetamide)

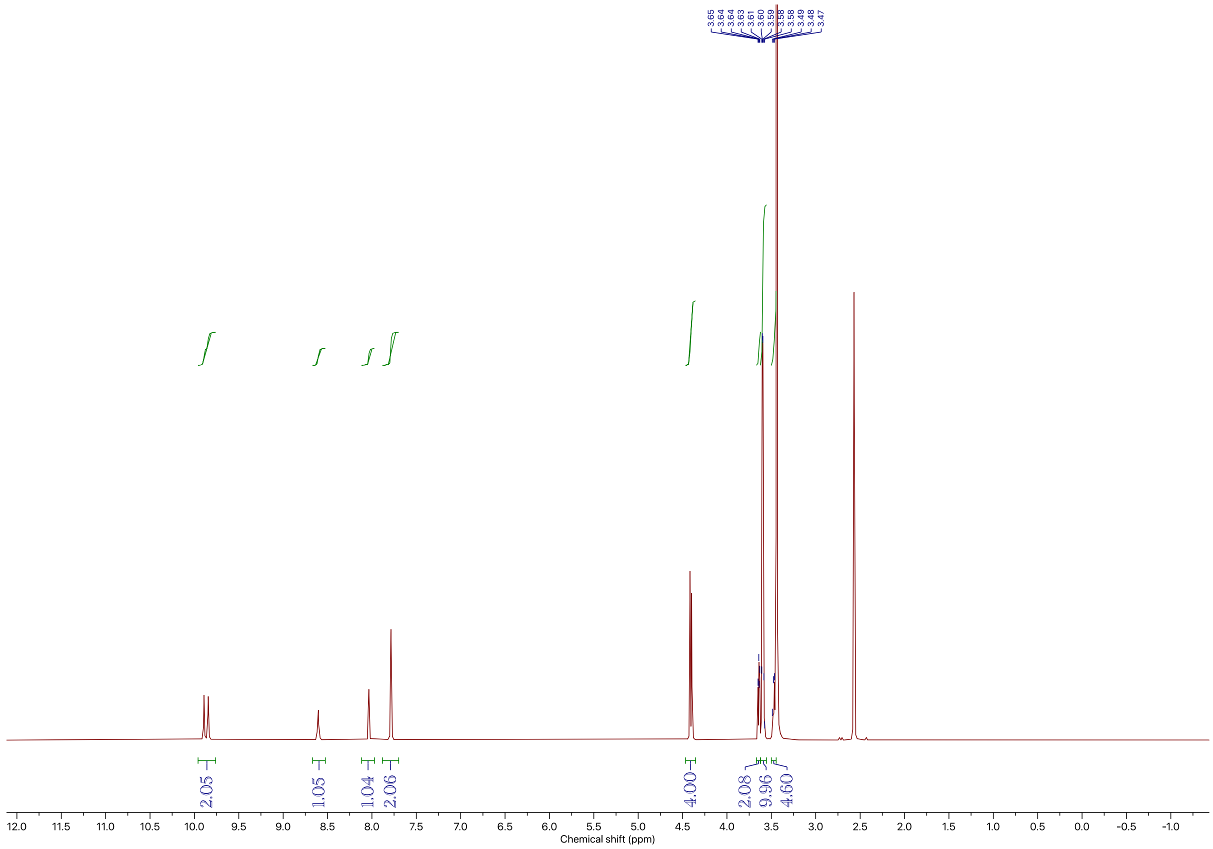

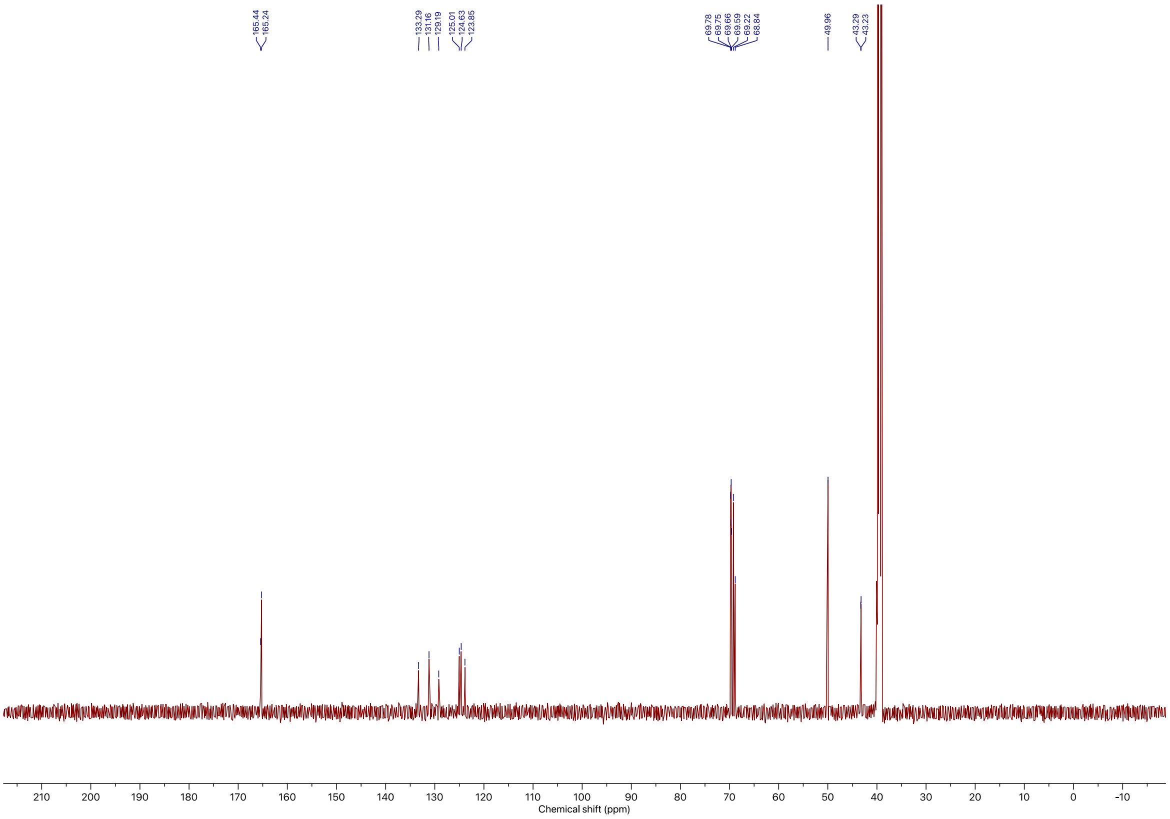
165.44
165.24
133.29
131.16
129.19
125.01
124.63
123.85
69.78
69.75
69.66
69.59
69.22
68.84
49.96
43.29
43.23
Chemical shift (ppm)

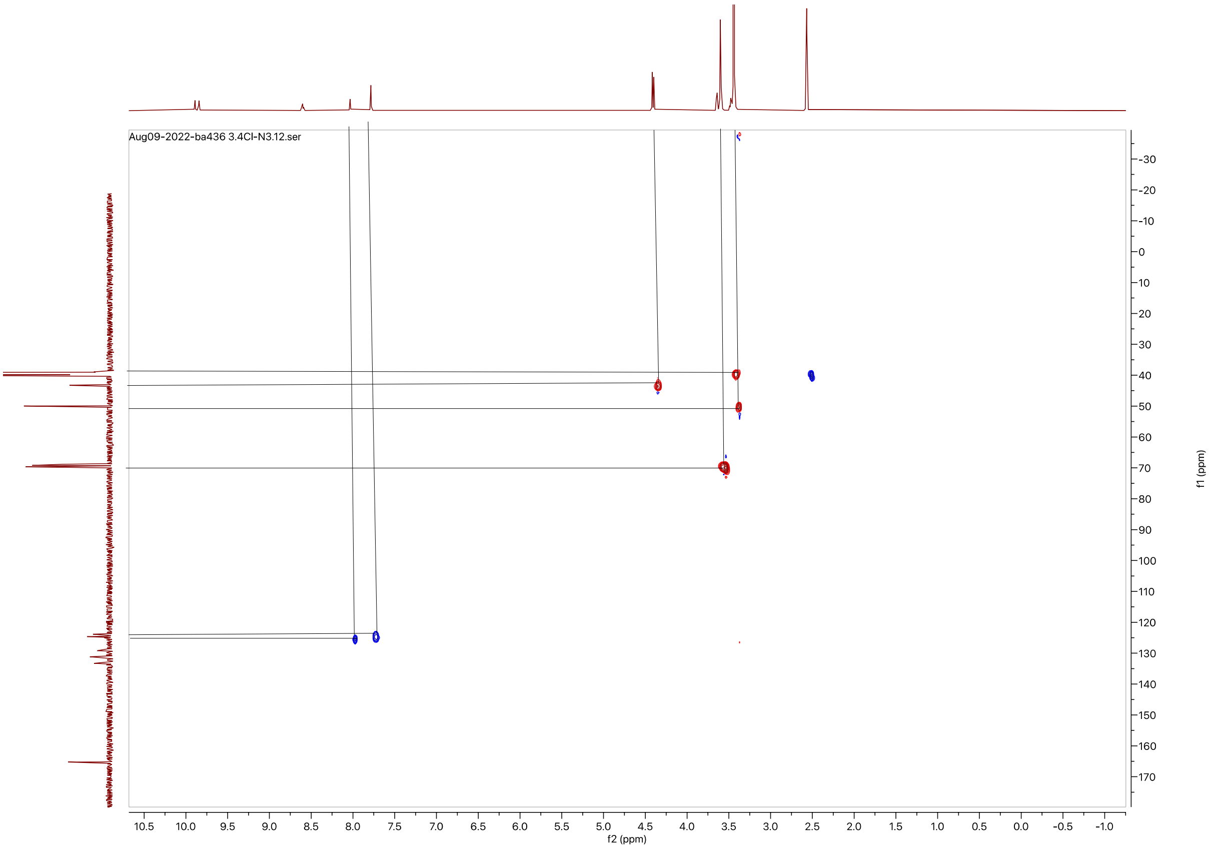
Aug09-2022-ba436 3.4Cl-N3.12.ser
f2 (ppm)
f1 (ppm)

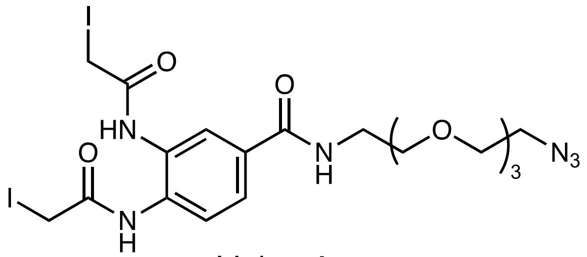

Linker **1**

N,*N'*-(4-((2-(2-(2-(2-azidoethoxy)ethoxy)ethoxy)ethyl)carbamoyl)-1,2-phenylene)bis(2-iodoacetamide)

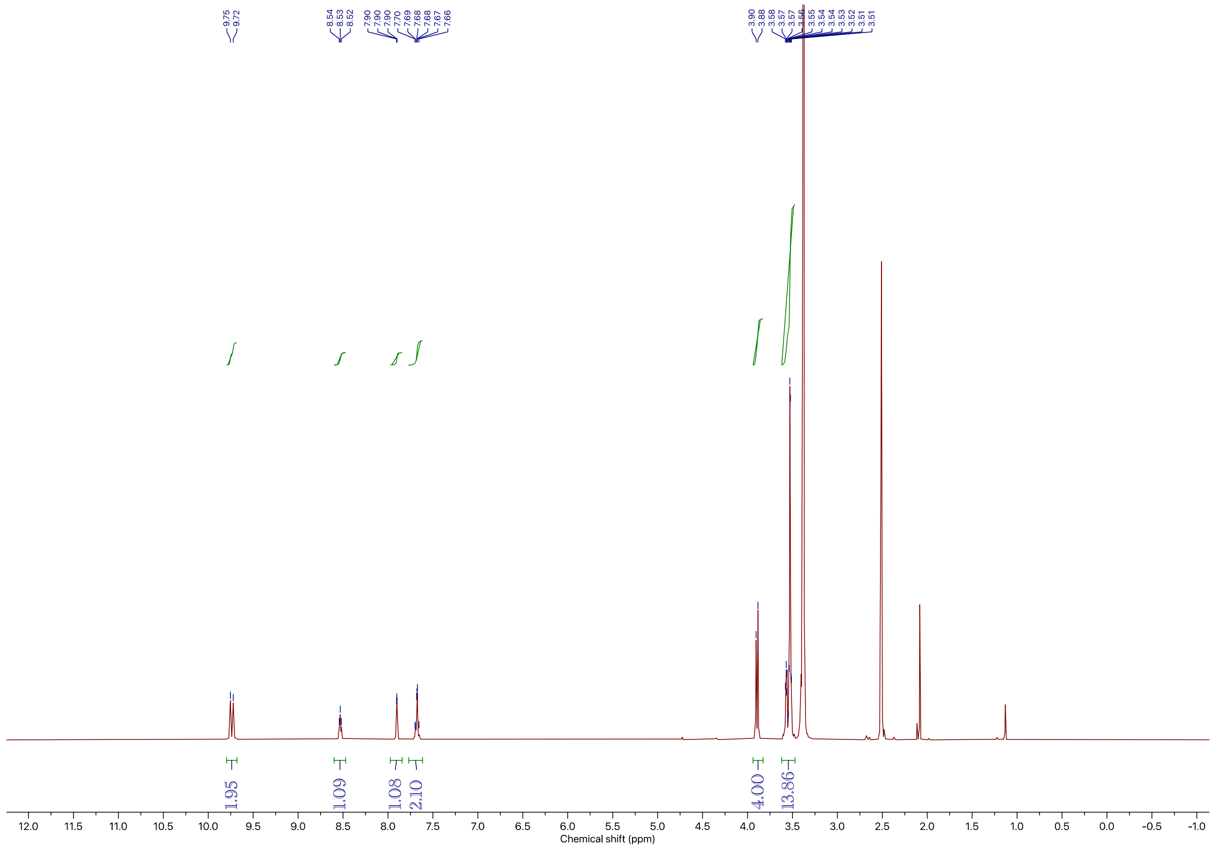

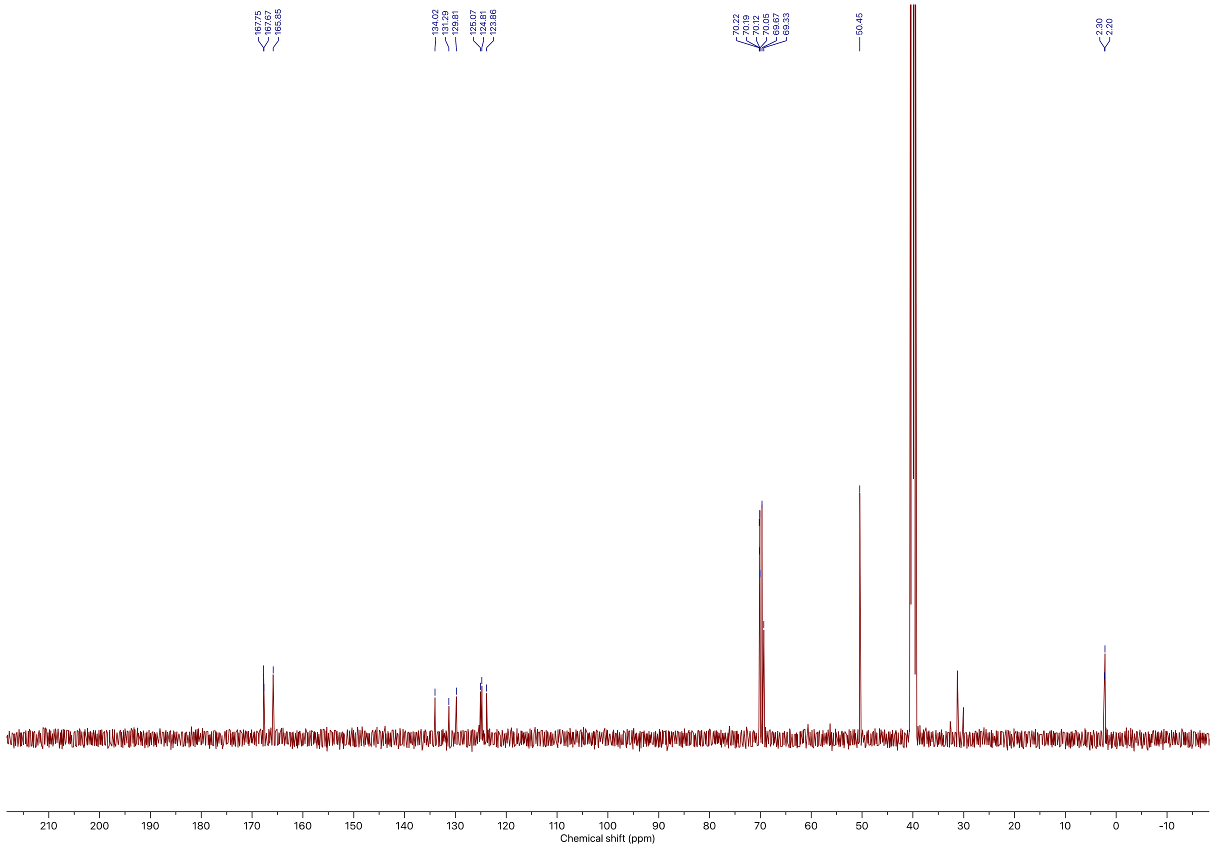
167.75
167.67
165.85
134.02
131.29
129.81
125.07
124.81
123.86
70.22
70.19
70.12
70.05
69.67
69.33
50.45
2.30
2.20
210 200 190 180 170 160 150 140 130 120 110 100 90 80 70 60 50 40 30 20 10 0 -10
Chemical shift (ppm)

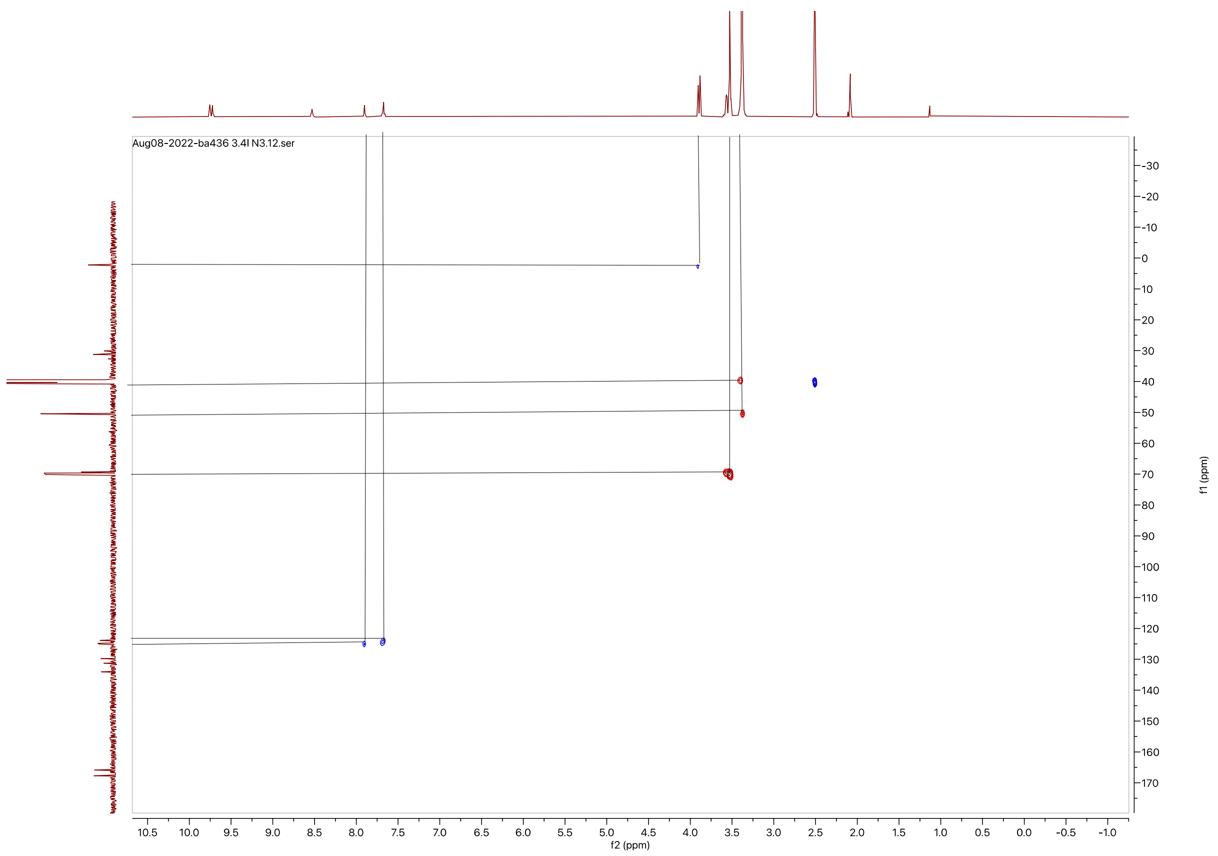
Aug08-2022-ba436 3.4I N3.12.ser
f2 (ppm)
f1 (ppm)

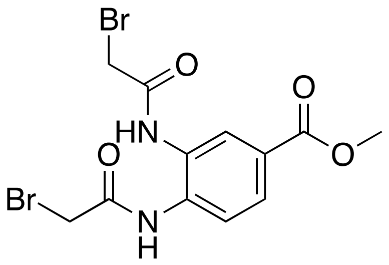

Linker **2**

methyl 3,4-bis(2-bromoacetamido)benzoate

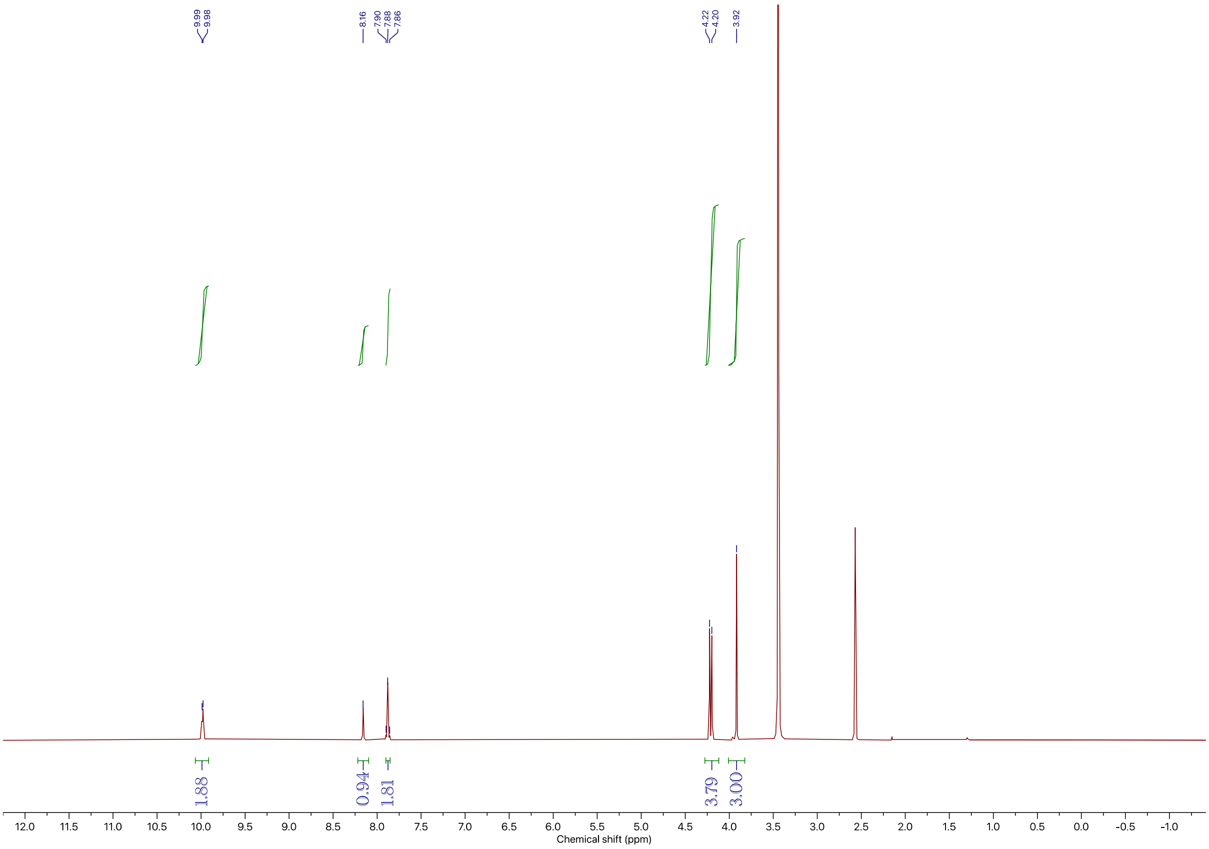

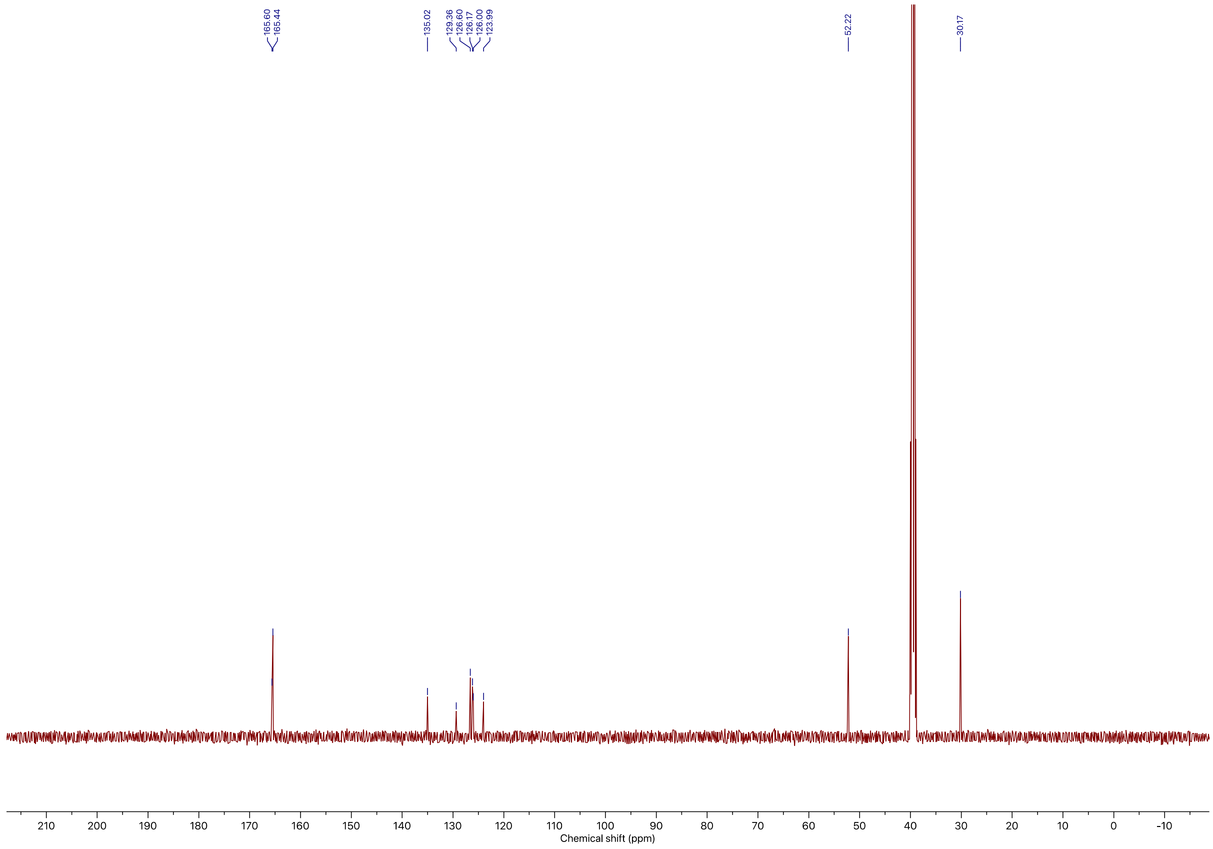
165.60
165.44
135.02
129.36
126.60
126.17
126.00
123.99
52.22
30.17
Chemical shift (ppm)

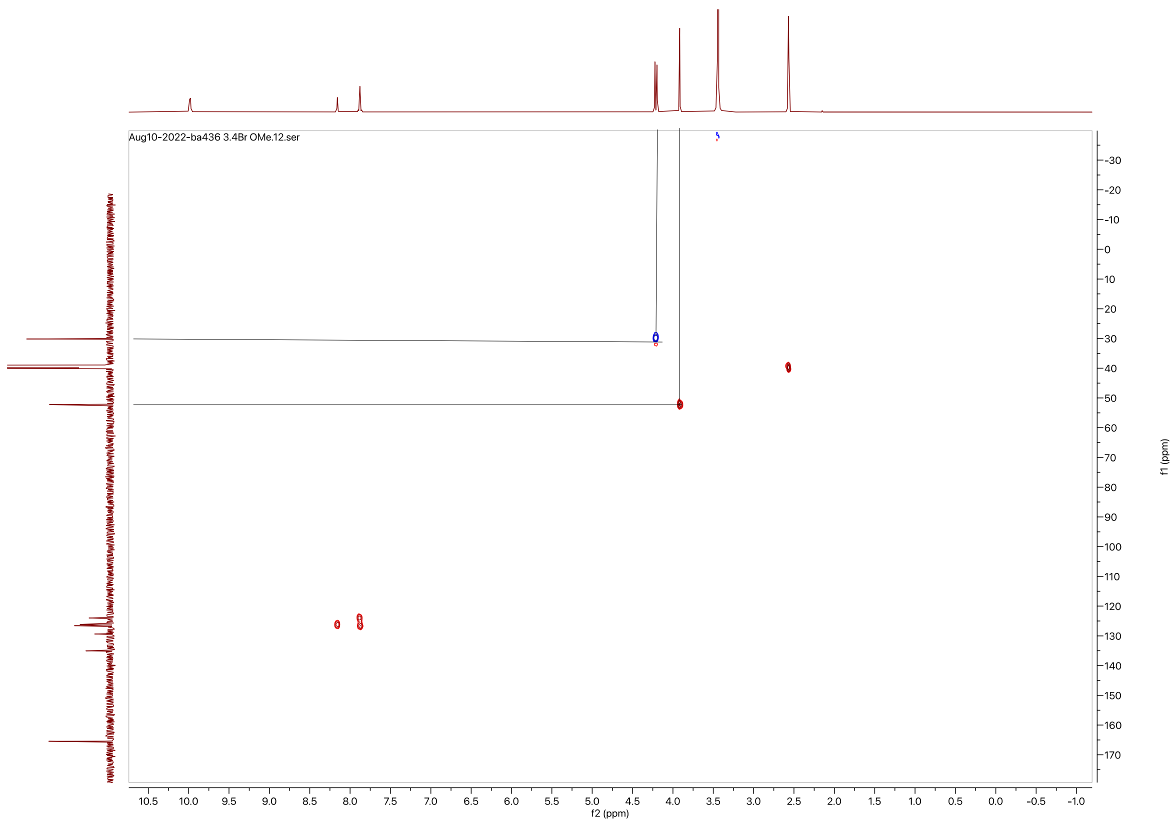
Aug10-2022-ba436 3.4Br OMe.12.ser
f2 (ppm)
f1 (ppm)

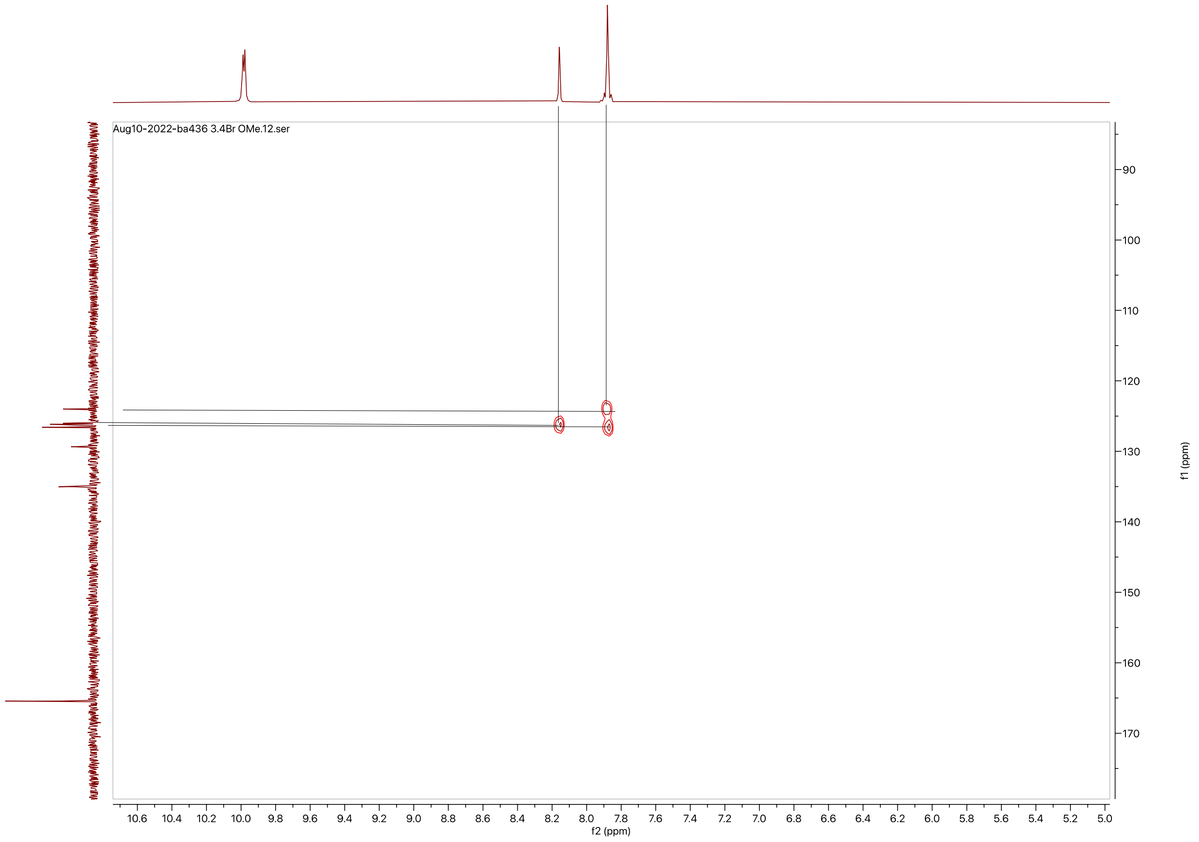
Aug10-2022-ba436 3.4Br OMe.12.ser
f2 (ppm)
f1 (ppm)

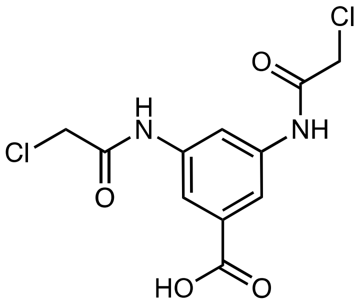

3,5-bis(2-chloroacetamido)benzoic acid

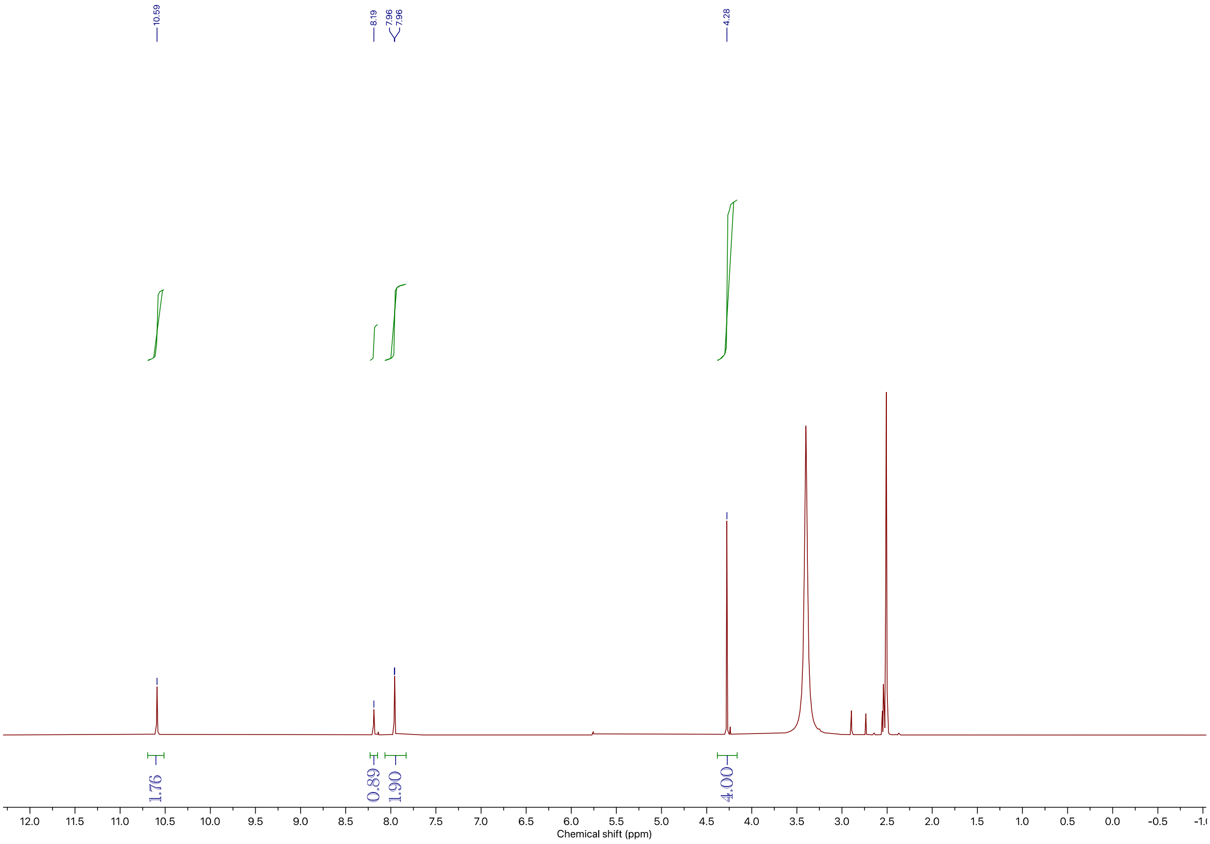

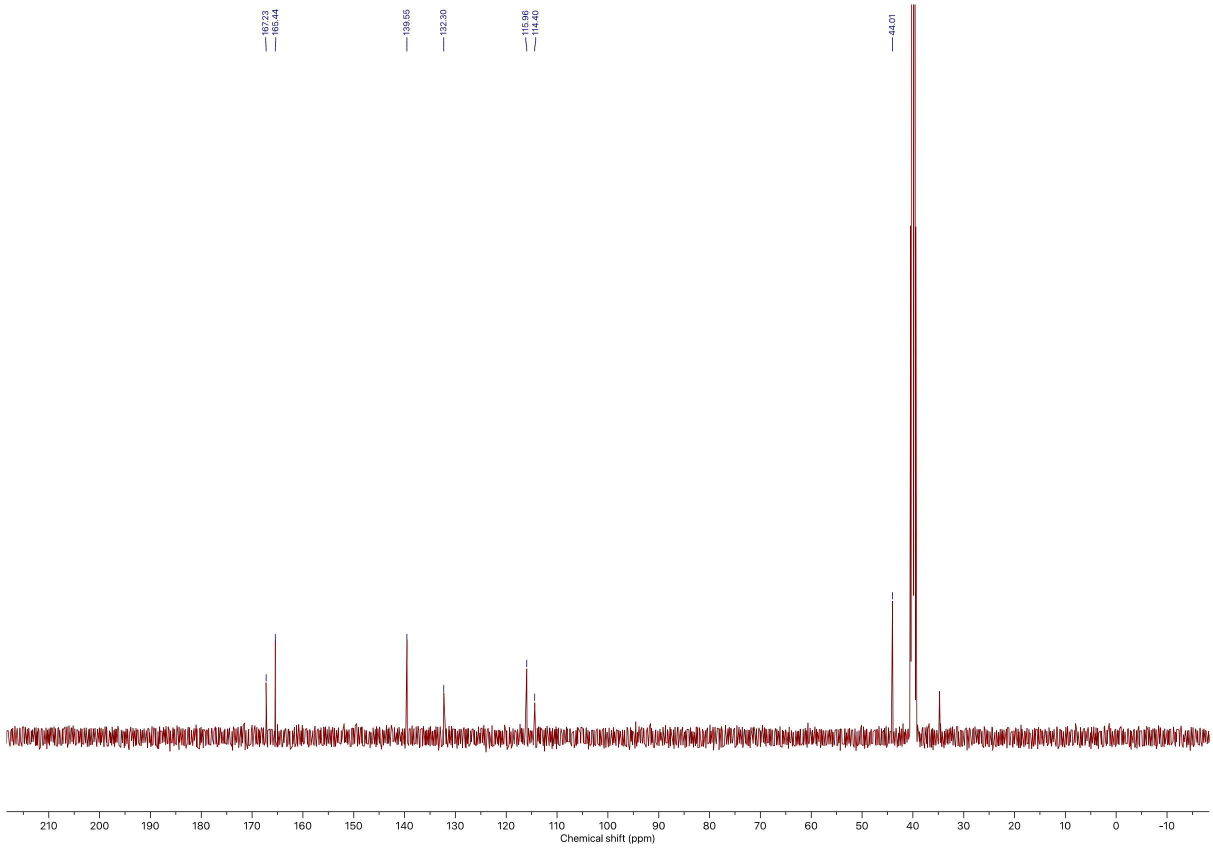
167.23
165.44
139.55
132.30
115.96
114.40
44.01
210 200 190 180 170 160 150 140 130 120 110 100 90 80 70 60 50 40 30 20 10 0 -10
Chemical shift (ppm)

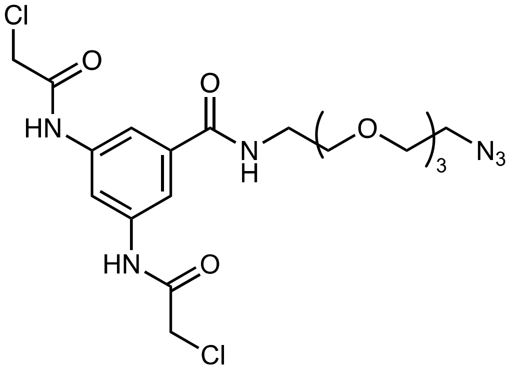

N,*N*'-(5-((2-(2-(2-(2-azidoethoxy)ethoxy)ethoxy)ethyl)carbamoyl)-1,3-phenylene)bis(2-chloroacetamide)

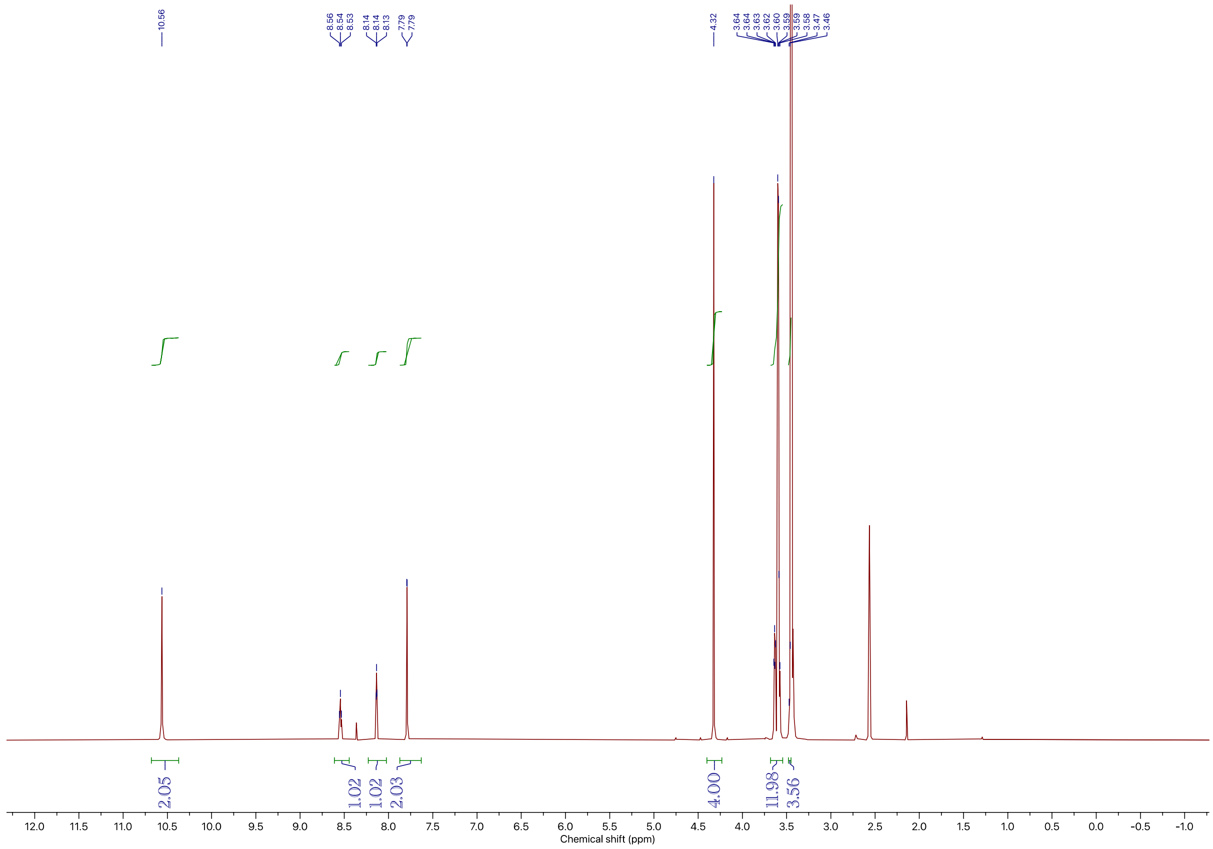

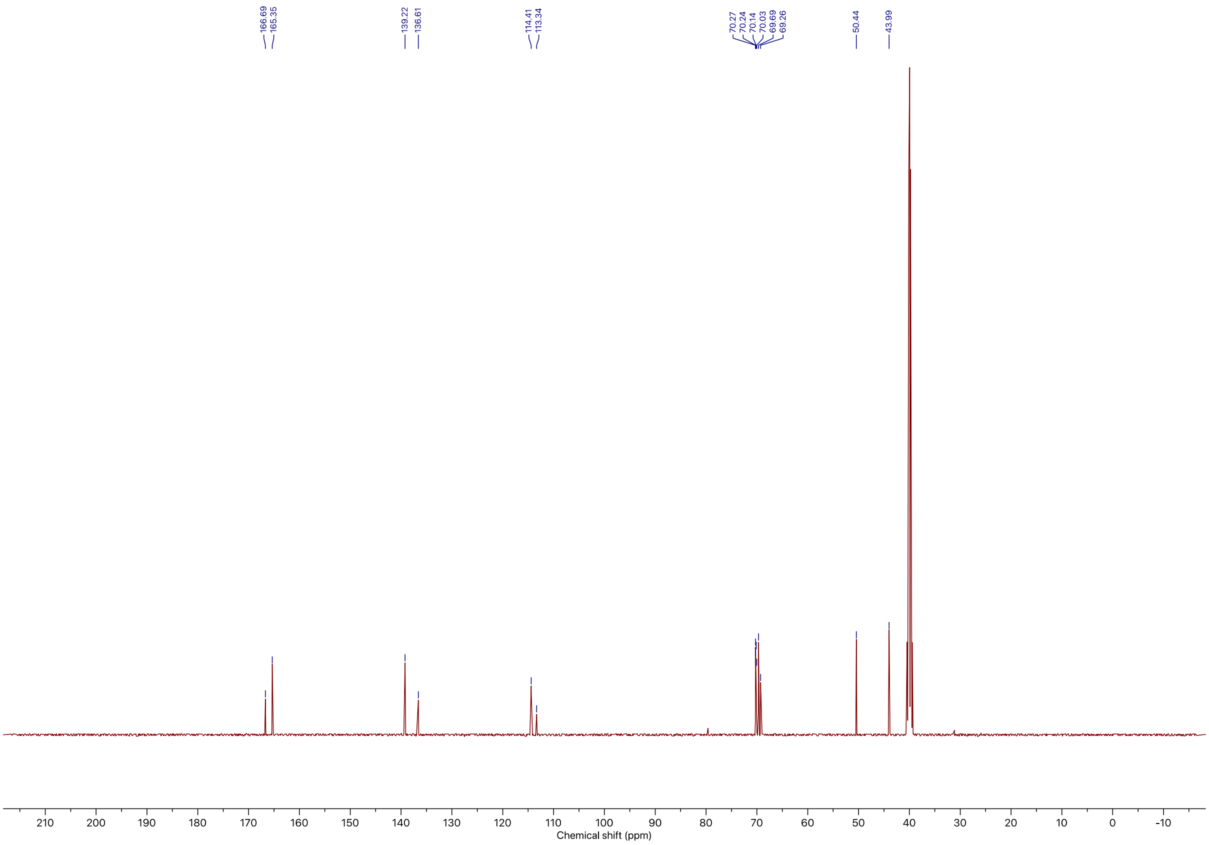
166.69
165.35
139.22
136.61
114.41
113.34
70.27
70.24
70.14
70.03
69.69
69.26
50.44
43.99
210 200 190 180 170 160 150 140 130 120 110 100 90 80 70 60 50 40 30 20 10 0 -10
Chemical shift (ppm)

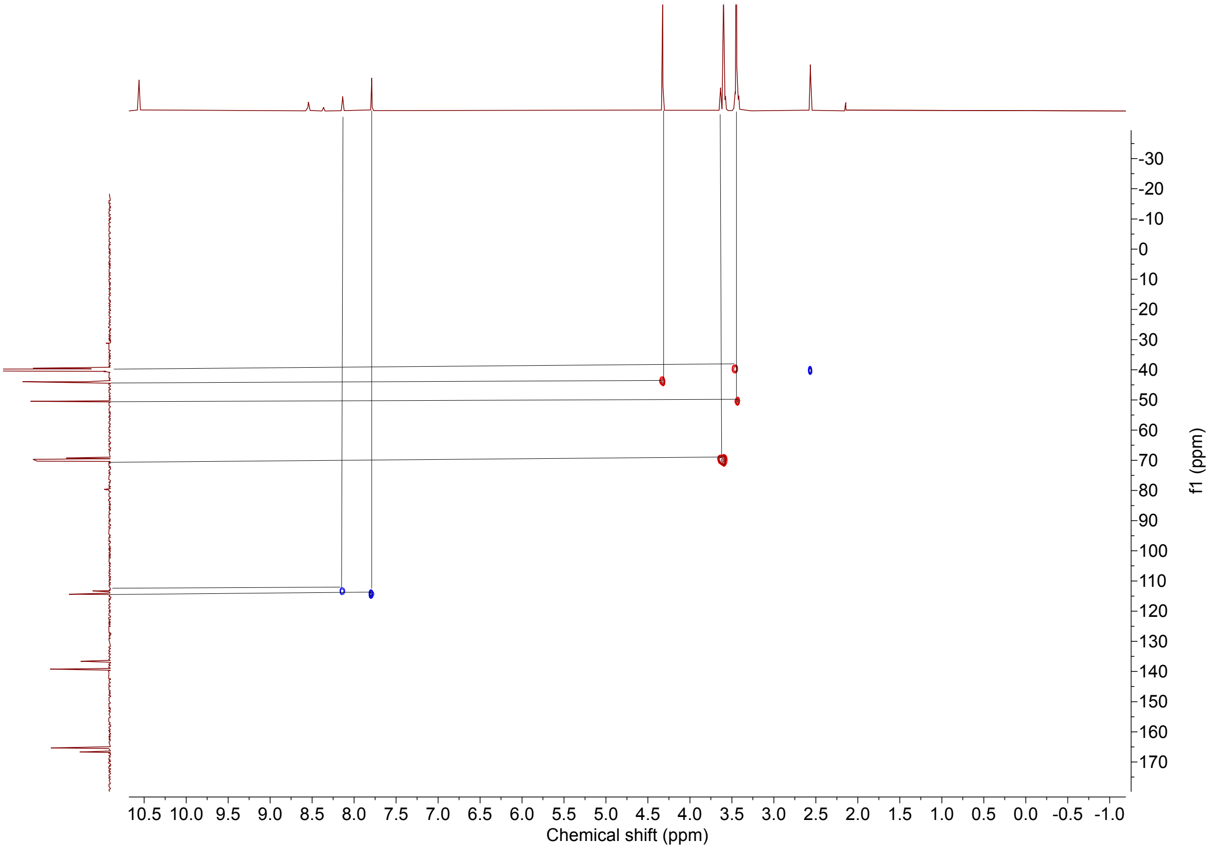
10.5 10.0 9.5 9.0 8.5 8.0 7.5 7.0 6.5 6.0 5.5 5.0 4.5 4.0 3.5 3.0 2.5 2.0 1.5 1.0 0.5 0.0 -0.5 -1.0
Chemical shift (ppm)
-30 -20 -10 0 10 20 30 40 50 60 70 80 90 100 110 120 130 140 150 160 170
f1 (ppm)

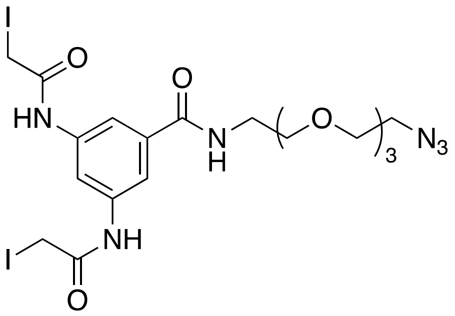

Linker **3**

N,N'-(5-((2-(2-(2-(2-azidoethoxy)ethoxy)ethoxy)ethyl)carbamoyl)-1,3-phenylene)bis(2-iodoacetamide)

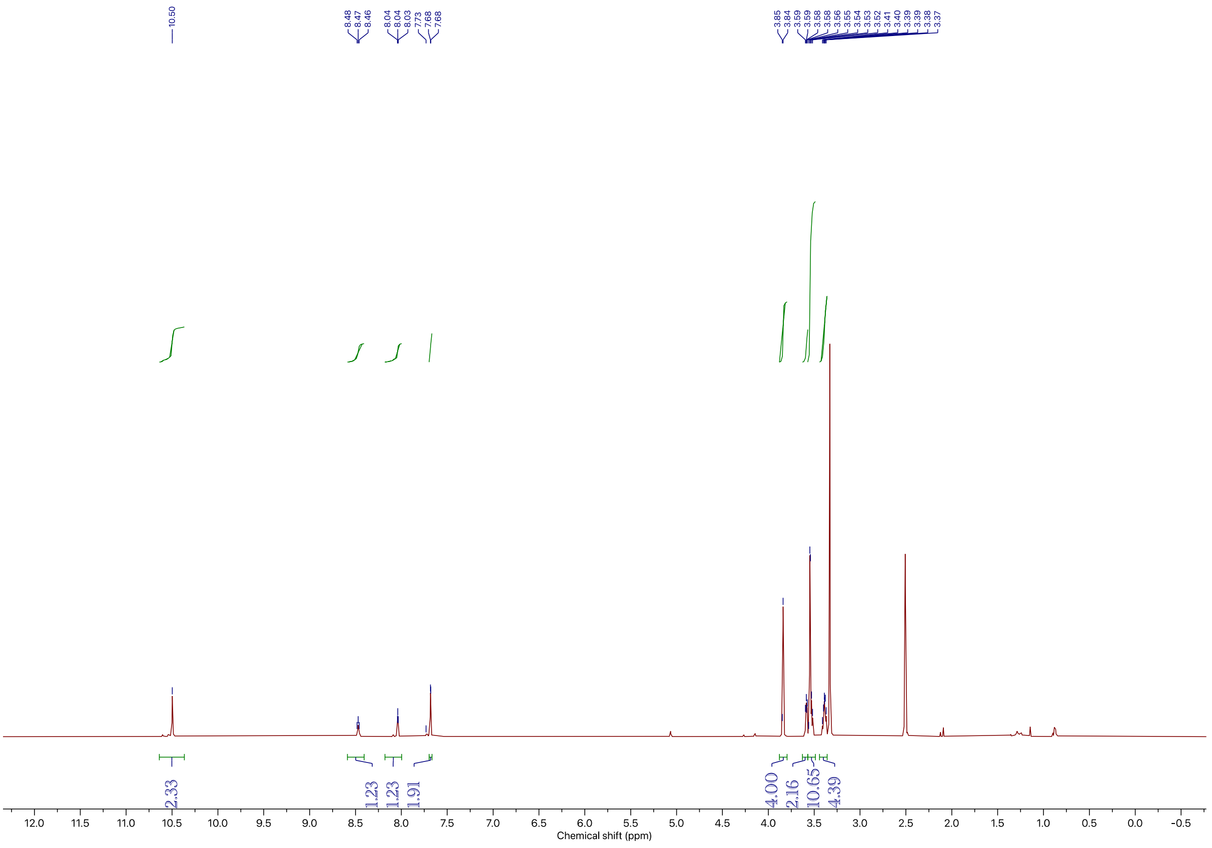

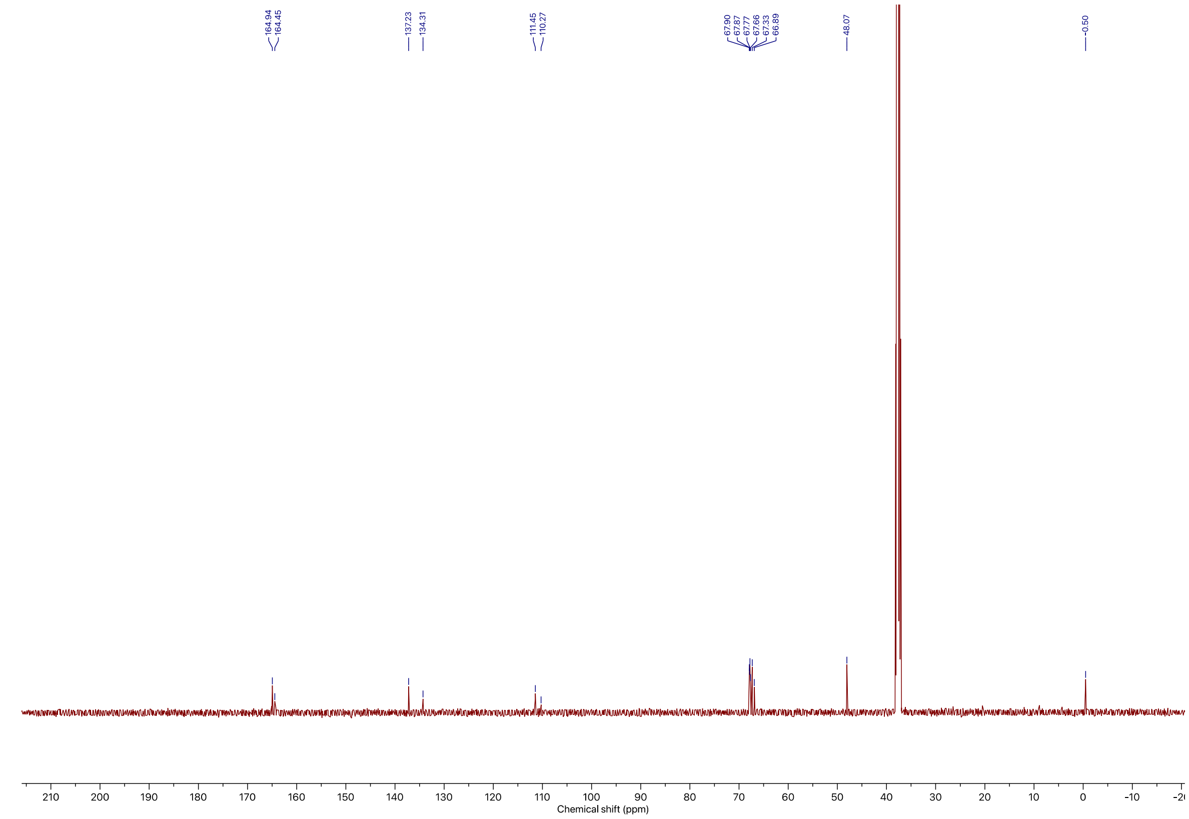
164.94
164.45
137.23
134.31
111.45
110.27
67.90
67.87
67.77
67.66
67.33
66.89
48.07
-0.50
Chemical shift (ppm)

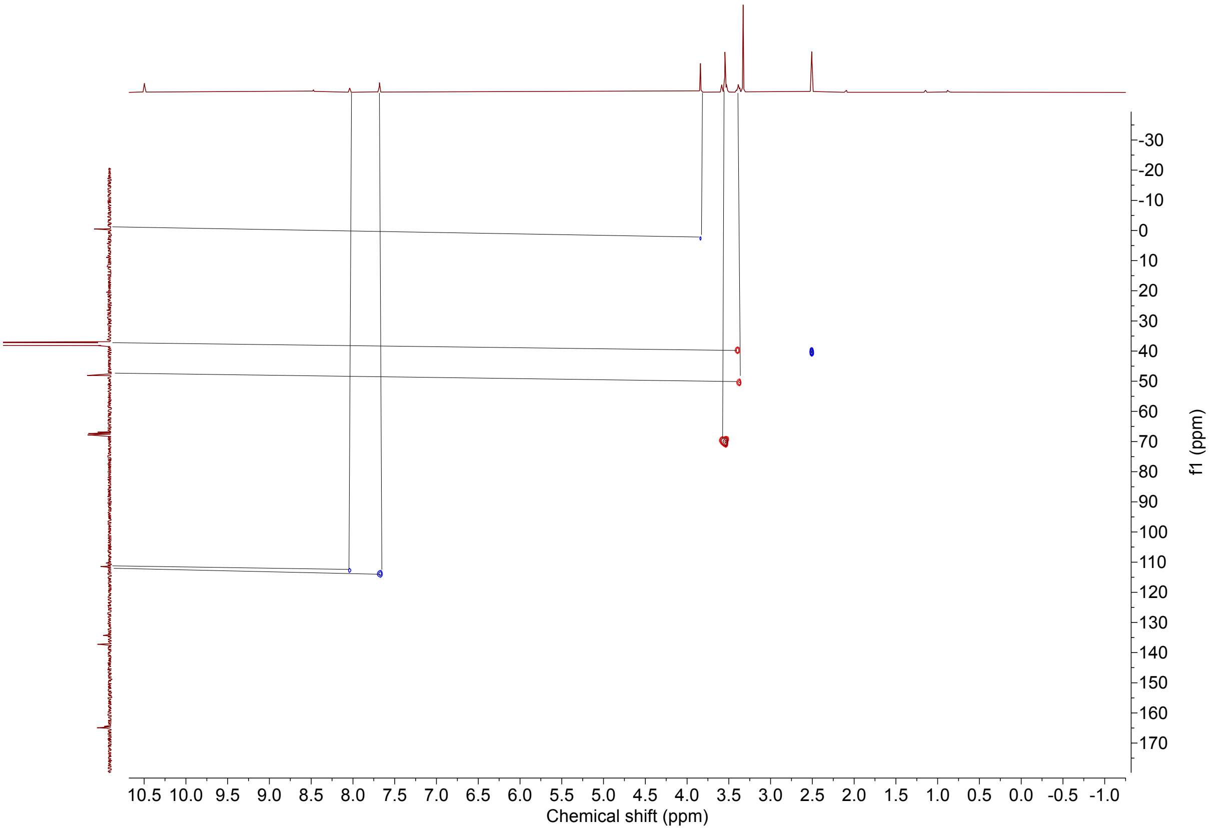
Chemical shift (ppm)
f1 (ppm)

References

(1) Lei, Q.; Wang, D.; Sun, K.; Wang, L.; Zhang, Y. Resistance Mechanisms of Anti-PD1/PDL1 Therapy in Solid Tumors. *Front. Cell Dev. Biol.* **2020**, *8*, 672. https://doi.org/10.3389/FCELL.2020.00672/BIBTEX.
